# SGLT2 inhibitors ameliorate NAFLD in mice *via* downregulating PFKFB3, suppressing glycolysis and modulating macrophage polarization

**DOI:** 10.1038/s41401-024-01389-3

**Published:** 2024-09-18

**Authors:** Xia-fang Lin, Xiao-na Cui, Jin Yang, Ya-fei Jiang, Tian-jiao Wei, Li Xia, Xin-yue Liao, Fei Li, Dan-dan Wang, Jian Li, Qi Wu, De-shan Yin, Yun-yi Le, Kun Yang, Rui Wei, Tian-pei Hong

**Affiliations:** 1 Department of Endocrinology and Metabolism, State Key Laboratory of Female Fertility Promotion, Peking University Third Hospital, Beijing, 100191 China; 2 State Key Laboratory of Vascular Homeostasis and Remodeling, Department of Endocrinology and Metabolism, Peking University Third Hospital, Beijing, 100191 China

**Keywords:** non-alcoholic fatty liver disease, dapagliflozin, canagliflozin, glycolysis, macrophage polarization, PFKFB3

## Abstract

Sodium-glucose co-transporter 2 (SGLT2) inhibitor (SGLT2i) is a novel class of anti-diabetic drug, which has displayed a promising benefit for non-alcoholic fatty liver disease (NAFLD). In this study, we investigated the protective effects of SGLT2i against NAFLD and the underlying mechanisms. The *db/db* mice and western diet-induced NAFLD mice were treated with dapagliflozin (1 mg·kg^−1^·d^−1^, i.g.) or canagliflozin (10 mg·kg^−1^·d^−1^, i.g.) for 8 weeks. We showed that the SGLT2i significantly improved NAFLD-associated metabolic indexes, and attenuated hepatic steatosis and fibrosis. Notably, SGLT2i reduced the levels of pro-inflammatory cytokines and chemokines, downregulated M1 macrophage marker expression and upregulated M2 macrophage marker expression in liver tissues. In cultured mouse bone marrow-derived macrophages and human peripheral blood mononuclear cell-derived macrophages, the SGLT2i (10, 20 and 40 μmol/L) significantly promoted macrophage polarization from M1 to M2 phenotype. RNA sequencing, Seahorse analysis and liquid chromatography-tandem mass spectrometry analysis revealed that the SGLT2i suppressed glycolysis and triggered metabolic reprogramming in macrophages. By using genetic manipulation and pharmacological inhibition, we identified that the SGLT2i targeted PFKFB3, a key enzyme of glycolysis, to modulate the macrophage polarization of M1 to M2 phenotype. Using a co-culture of macrophages with hepatocytes, we demonstrated that the SGLT2i inhibited lipogenesis in hepatocytes *via* crosstalk with macrophages. In conclusion, this study highlights a potential therapeutic application for repurposing SGLT2i and identifying a potential target PFKFB3 for NAFLD treatment.

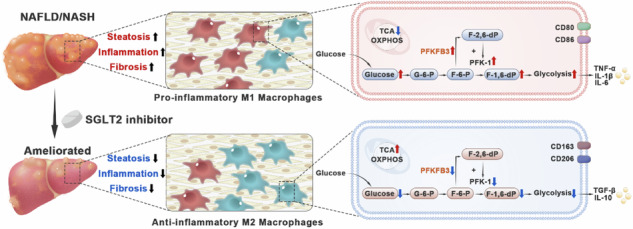

## Introduction

Non-alcoholic fatty liver disease (NAFLD) is a spectrum of diseases, which includes diffuse non-alcoholic hepatic steatosis, non-alcoholic steatohepatitis (NASH), liver cirrhosis and hepatocellular carcinoma, and has already been a global health threat [1]. Around 25% of the overall population is estimated to have NAFLD, making it a main cause of chronic liver illness in the world [2]. NAFLD is characterized by the occurrence of steatosis in over 5% of liver cells, excluding the known factors such as excessive alcohol intake, medications, or other long-term liver disorders [3]. However, the pathogenesis of NAFLD is intricate and still not fully comprehended.

Macrophages play an important role in the maintenance of organ function and homeostasis. Traditionally, macrophages are divided into two main subpopulations: classically activated pro-inflammatory macrophages (M1) and alternatively activated anti-inflammatory macrophages (M2) [4, 5]. M1 macrophages activated by lipopolysaccharide (LPS) alone or in combination with interferon-γ (IFN-γ) exhibit pro-inflammatory characteristics, and release cytokines and chemokines, such as interleukin (IL)-1β, IL-6, tumor necrosis factor-α (TNF-α) and C-C motif chemokine ligand-2 (CCL-2). In contrast, IL-4/IL-13-activated M2 macrophages, play a significant role in inflammation resolution and wound healing, with IL-10 and transforming growth factor-β (TGF-β) production [5]. Liver macrophages, including Kupffer cells (resident macrophages in liver) and monocyte-derived macrophages (originated from bone marrow), contribute to the pathogenesis of NAFLD [6, 7]. Therefore, induction of macrophage phenotype shift would be a potential approach for NAFLD treatment. However, whether and how this phenotype shift could be induced in NAFLD remains largely unexplored.

Sodium-glucose co-transporter 2 (SGLT2) inhibitor (SGLT2i), including dapagliflozin, empagliflozin and canagliflozin, is a new class of anti-diabetic drug. Recently, SGLT2i has revealed strong anti-inflammatory and antioxidant properties, which positions it as potential candidate for NAFLD treatment [8]. In type 2 diabetic patients with NAFLD, a 20-week empagliflozin treatment reduced liver fat content, with lowered liver enzyme levels [9]. Similarly, a 24-week canagliflozin treatment improved hepatic histology, with decreased scores of hepatic steatosis, lobular inflammation, ballooning and fibrosis [10]. Besides, SGLT2i polarized M2 macrophages in white adipose tissues, thereby attenuating obesity-induced inflammation and insulin resistance in diet-induced obese mice [11]. Nevertheless, how SGLT2i induced M1 to M2 phenotype shift remained unknown.

In this study, we aimed to clarify the protective role and underlying mechanism of SGLT2i in NAFLD. The effects of dapagliflozin and canagliflozin on NAFLD were investigated in mouse NAFLD models, and their impacts on macrophage polarization were evaluated in the liver tissues of NAFLD mice and in cultured mouse and human macrophages. Subsequently, the involved factors were explored using RNA sequencing (RNA-seq), Seahorse analysis and liquid chromatography-tandem mass spectrometry (LC-MS/MS), and 6-phosphofructo-2-kinase/fructose-2,6-bisphosphatase (PFKFB) 3, a key enzyme of glycolysis, was identified as a potential mediator. By using genetic manipulation, pharmacological inhibition, and co-culture of macrophages with hepatocytes, the involvement of PFKFB3 in promoting the macrophage polarization of M1 to M2 phenotype and inhibiting lipogenesis in hepatocytes was confirmed. Our study highlights a potential therapeutic strategy for repurposing SGLT2i as an approach for NAFLD treatment.

## Materials and methods

### Animals and intervention

All animal experimental procedures were approved by Peking University Animal Care and Use Committee (LA2020508). Animals were housed conventionally with free access to food and water on a 12-h light/dark cycle.

Seven-week-old male *db/db* (BKS-*Lepr*^em2Cd479^/Gpt) mice (GemPharmatech, Nanjing, China), a NAFLD model [12, 13], were adaptively fed for one week, and weight-matched animals were randomly assigned to three groups (*n* = 9 per group). Dapagliflozin (1 mg·kg^−1^·d^−1^, HY-10450; MedChemExpress, Monmouth Junction, NJ, USA), canagliflozin (10 mg·kg^−1^·d^−1^, HY-10451; MedChemExpress) or vehicle were administered intragastrically daily for 8 weeks. The doses of dapagliflozin and canagliflozin were referred to the previous studies [14–17]. Vehicle-treated male *db/m* littermates were used as normal control (*n* = 10).

Six-week-old male C57BL/6J mice (GemPharmatech) were randomized into chow diet (CD) feeding (*n* = 6) and western diet (WD) feeding (*n* = 45). Mice were fed for 20 weeks on a CD (fat 10%, carbohydrate 70% and protein 20%; Research Diets, New Brunswick, NJ, USA) and on a high-fat, high-fructose and high-cholesterol diet (palm oil 40%, fructose 20% and cholesterol 2%; Research Diets), respectively [12, 13]. WD-fed mice weighing over 40 g were chosen for further experiments (*n* = 20) [18, 19], and divided into three groups (*n* = 6−7 per group). For the weight-matched WD-fed mice, dapagliflozin (1 mg·kg^−1^·d^−1^), canagliflozin (10 mg·kg^−1^·d^−1^) or vehicle were administered intragastrically daily for 8 weeks. Vehicle-treated CD-fed mice served as normal control (*n* = 6).

Since global homozygous disruption of *Pfkfb3* results in embryonic lethality before E8 [20], global heterozygous *Pfkfb3* knockout (*Pfkfb3*^+/−^) mice were usually used for the experiments [21–24]. *Pfkfb3*^+/−^ mice were generated on a C57BL/6J genetic background by GemPharmatech. Briefly, CRISPR/Cas9 technology was used to modify *Pfkfb3* gene and caused a knockout of the 223-bp coding sequence in exon2-exon3 of *Pfkfb3*. Six-week-old male *Pfkfb3*^*+/−*^ mice and littermate *Pfkfb3*^*+/+*^ mice (*n* = 6 per group) were fed on a WD for 12 weeks that were used for inducing metabolic inflammation [25].

Six-week-old male C57BL/6J mice were fed on a WD for 8 weeks that were used for inducing metabolic inflammation [25]. WD-fed mice were divided into two groups (*n* = 6 per group) and were treated for 4 weeks with the PFKFB3-specific inhibitor 3-(3-pyridinyl)-1-(4-pyridinyl)-2-propen-1-one (3PO; 50 mg/kg, 4 times every week, HY-19824; MedChemExpress) or vehicle.

### Histological staining

For H&E, Masson and Sirius red staining, liver tissues were fixed in a 4% (*w*/*v*) paraformaldehyde solution overnight, and then embedded in paraffin and cut into 5-µm sections. For oil red O staining, liver tissues were cryopreserved in O.C.T. compound (Tissue-Tek; Torrance, CA, USA) and prepared into 10-µm sections. Sections were subjected to staining by using H&E, Masson, Sirius red and oil red O staining kits (all from Solarbio, Beijing, China) following the manufacturer’s instructions. The stained sections were analyzed using a digital scanner (NanoZoomer-SQ; Hamamatsu, Hamamatsu City, Japan). The kits are summarized in Supplementary Table S1.

### Immunofluorescent staining

Liver frozen sections were prepared in the same way as for oil red O staining. Sections were blocked with goat serum and incubated with primary antibodies at 4 °C overnight. Subsequently, sections were incubated with secondary antibodies for 1 h at room temperature. Finally, sections were stained with DAPI. The fluorescence was visualized with a Leica TCS SP8 confocal fluorescence microscope (Leica Microsystems, Wetzlar, Germany). The specific information of all antibodies is listed in Supplementary Table S2.

### Liver lipid, cytokine and chemokine analyses

For each mouse, liver sample from the same region of left lobe was collected for the following detection. Liver tissues (100 mg) were homogenized in radioimmunoprecipitation assay lysis buffer (Applygen, Beijing, China). Biochemical assay kits for triglyceride (TG) and total cholesterol (TC) (both from Applygen), and specific ELISA kits for mouse TNF-α, IL-1β, IL-6, CCL-2, IL-10 and TGF-β (all from Invitrogen, Carlsbad, CA, USA) were used to detect the lipids, cytokines and chemokines in liver tissue lysates in accordance with the manufacturer’s instructions. The specific information of all kits is listed in Supplementary Table S1.

### Culture and intervention of primary mouse bone marrow-derived macrophages (BMDMs)

To obtain murine BMDMs, the femurs of C57BL/6 mice aged 6−10 weeks were dissected, the bone ends were removed, and bone marrow was flushed from femurs with sterile phosphate buffered saline (PBS). Bone marrow cells were collected by centrifugation in DMEM (Gibco, Grand Island, NY, USA) at 400 × *g* for 4 min. Subsequently, cells were suspended in a complete DMEM medium containing 10% fetal bovine serum (FBS; Gibco), 20 ng/mL recombinant macrophage colony-stimulating factor (M-CSF; PeproTech, Rocky Hill, NJ, USA) and 1% penicillin-streptomycin (Gibco) in petri dish at a density of 1 ×10^6^ cell/mL, and the day was marked as day 0. On day 3, half of the medium was replaced with fresh medium. By day 7, cells were deemed as naïve macrophages and prepared for the experiments. BMDMs were cultured for 12 h with dapagliflozin (10, 20 and 40 μmol/L) or canagliflozin (10, 20 and 40 μmol/L) or vehicle in the presence or absence of LPS (100 ng/mL; Sigma-Aldrich, St. Louis, MO, USA) + IFN-γ (50 ng/mL; PeproTech).

### Culture and intervention of primary human peripheral blood mononuclear cell (PBMC)-derived macrophages

PBMCs were collected from patients with type 2 diabetes. Written informed consent was obtained from blood donors in accordance with ethical approval from the Ethics Committee of Peking University Third Hospital (M2022039).

PBMCs were separated using Ficoll gradient centrifugation. Briefly, blood was diluted at 1:1 in PBS. Ficoll solution (5 mL; Sigma-Aldrich) was added to a 15-mL centrifuge tube and the diluted blood (10 mL) was softly added on the top of Ficoll solution. The tube was centrifuged at 700−800 × *g* for 20−30 min at 4 °C (no-brake). PBMCs were obtained from the interphase and rinsed twice with PBS. Subsequently, PBMCs were purified by using magnetic bead sorting with MojoSort™ human CD14 selection kits (Biolegend, San Diego, CA, USA). The kits are listed in Supplementary Table S1.

Purified PBMCs were suspended in RPMI medium (Gibco) with 25 ng/mL M-CSF, 10% FBS and 1% penicillin-streptomycin at a density of 1 ×10^6^ cell/mL, and pre-incubated for 6 d. On day 3, half of the medium was replaced by fresh medium. The PBMC-derived macrophages were cultured for 12 h on day 7 with 20 μmol/L dapagliflozin, canagliflozin or vehicle in the presence or absence of LPS (100 ng/mL) + IFN-γ (50 ng/mL).

### Culture and intervention of mouse macrophage and hepatocyte cell lines

Mouse macrophage cell line RAW264.7 cells (Procell, Wuhan, China) were maintained in DMEM supplemented with 10% FBS and 1% penicillin-streptomycin, and 2.5 ×10^5^ cells were placed in each well of 6-well plates for seeding. After a 12-h pre-incubation, RAW264.7 cells were cultured for 12 h with 20 μmol/L dapagliflozin, canagliflozin or vehicle in the presence or absence of LPS (100 ng/mL).

Mouse hepatocyte cell line AML-12 cells (Procell) were incubated in DMEM/F12 (Gibco) containing 10% FBS, 10 μg/mL insulin, 5.5 μg/mL transferrin, 40 ng/mL dexamethasone, 5 ng/mL selenium and 1% penicillin-streptomycin (all from Gibco), and 2 ×10^5^ cells were placed in each well of 6-well plates for seeding. After a 24-h pre-incubation, AML-12 cells were cultured for 24 h with 20 μmol/L dapagliflozin, canagliflozin or vehicle in the presence or absence of palmitic acid (PA; 100 μmol/L; Sigma-Aldrich) + oil acid (OA; 200 μmol/L; Sigma-Aldrich) that were used for inducing an in vitro model of liver lipid accumulation.

### Co-culture of mouse macrophage and hepatocyte cell lines

Transwell chamber with 0.4-μm pores (Corning, New York, NY, USA) was used to establish the co-culture system. RAW264.7 cells were seeded in top chamber and pre-incubated for 12 h with 20 μmol/L dapagliflozin, canagliflozin or vehicle in the presence or absence of LPS (100 ng/mL). AML-12 cells were seeded in bottom chamber and pre-incubated for 24 h with PA (100 μmol/L) + OA (200 μmol/L). Two types of cells were pre-incubated separately. Before co-culturing, cells were rinsed with PBS and new culture media without the above agents were replaced. After a 24-h incubation in the co-culture systems, AML-12 cells were harvested for subsequent analysis.

### ELISA and chemical assays

Specific ELISA kits for mouse TNF-α, IL-1β, IL-6, CCL-2 and IL-10, and human TNF-α, IL-6 and CCL-2 (all from Invitrogen) were used to measure the cytokines and chemokines in cell-free culture supernatants according to the manufacturer’s instructions. Nitric oxide was measured by using a chemical assay kit (Beyotime, Shanghai, China) in accordance with the manufacturer’s instructions. The specific information of all kits is summarized in Supplementary Table S1.

### Flow cytometry

Cells were collected, rinsed in cold PBS containing 1% FBS, and centrifuged at 400 × *g* for 5 min at 4 °C. The supernatant was discarded and the cells were resuspended in PBS, followed by addition of required antibodies against macrophage markers in accordance with the manufacturer’s recommendation. The antibodies are summarized in Supplementary Table S2. After vortexed for 5 s, cells were incubated for 30 min at 4 °C in the dark. Cells were rinsed twice with PBS, centrifuged at 400 × *g* for 5 min, and then resuspended in 100 μL PBS. The cells were quickly analyzed by a flow cytometer (CytoFlex; Beckman Coulter, Miami, FL, USA). Data were analyzed by using CytExpert 2.4 software (Beckman Coulter) and FlowJo 7.6.1 software (FlowJo LLC, Ashland, OR, USA).

### RNA-seq and data analysis

To examine the effect of SGLT2i on macrophage transcriptome, BMDMs were cultured for 12 h with 20 μmol/L dapagliflozin, canagliflozin or vehicle in the presence or absence of LPS (100 ng/mL) and IFN-γ (50 ng/mL). Four sets of BMDMs were harvested for extracting total RNA with TRIzol reagent (Thermo Fisher Scientific, Waltham, MA, USA) in accordance with the manufacturer’s instructions. PCR was used to amplify cDNA fragments following reverse transcription. Illumina Genome Analyzer II system was utilized for RNA-seq (MetWare Biotechnology Co., Ltd., Wuhan, China). Gene expression was quantified with fragments per kilobase of transcript per million mapped reads. Differentially expressed genes (DEGs) were determined using R software by identifying genes with *P*-value < 0.01 and fold change >1.5 or <0.666. Enriched biological processes were analyzed by using Kyoto Encyclopedia of Genes and Genomes (KEGG) pathway enrichment analysis.

### Seahorse analysis

Extracellular acidification rate (ECAR) and oxygen consumption rate (OCR), indicative of glycolysis and respiration respectively, were monitored using an XF96 Extracellular Flux Analyzer (Agilent Technologies, Santa Clara, CA, USA) with Seahorse XF glycolytic rate assay kit and Seahorse XF cell Mito stress test kit (both from Agilent Technologies), respectively. The kits are listed in Supplementary Table S1. Briefly, BMDMs were suspended in phenol red-free DMEM media supplemented with 10 mmol/L glucose, 2 mmol/L glutamine and 1 mmol/L pyruvate. Cells were placed into a 96-well Fluxpak (Agilent Technologies) at a density of 1 ×10^4^ cells per well, and then cultured for 12 h with 20 μmol/L dapagliflozin, canagliflozin or vehicle in the presence or absence of LPS (100 ng/mL) + IFN-γ (50 ng/mL). Mitochondrial respiratory parameters and glycolytic parameters were assessed by OCR (pmol/min) and ECAR (mpH/min), respectively using the injection solutions: oligomycin, carbonyl cyanide 4-(trifluoromethoxy) phenylhydrazone, rotenone/antimycin A, and 2-deoxy-D-glucose.

### Glucose uptake assay

BMDMs were equilibrated in PBS with 1% FBS for 30 min. Cells were cultured for 30 min at 37 °C in PBS containing 100 μmol/L 2-[N-(7-nitrobenz-2-oxa-1,3-diazol-4-yl) amino]-2-deoxy-D-glucose (Invitrogen) with 20 μmol/L dapagliflozin or canagliflozin or vehicle in the presence or absence of LPS (100 ng/mL) + IFN-γ (50 ng/mL). The cells were washed twice by PBS and the flow cytometry analysis was carried out using a flow cytometer (CytoFlex).

### Metabolite detection by LC-MS/MS

Intracellular metabolite detection was conducted using LC-MS/MS as previously described [26]. ACQUITY UPLC-I-Class was coupled to Xevo TQ-S micro-ESI tandem mass system (Waters, Milford, MA, USA) for metabolite separation and detection. A reversed-phase chromatography method with an Agilent Poroshell 120 SB-Aq column (2.1 mm × 100 mm, 2.7 μm) was used for compound separation at 30 °C. Mobile phase used ultrapure water containing 5 mmol/L ammonium acetate and acetonitrile in A phase and B phase, respectively. Liquid chromatography gradient was listed as follows: the starting condition was 100% A phase, then the proportion of B phase gradually increased to 50% from 0.5 min to 4 min, and flushing ratio was subsequently stable with 100% B phase from 4 min to 6 min. At 6 min, the proportion of mobile phase returned to the initial proportion, and balanced for 2 min. The flow rate was 0.3 mL/min and inject volume was 2 μL for electrospray ionization (ESI)+ and 5 μL for ESI−.

Triple quadrupole mass spectrometer was equipped with ESI ion source, and the relevant parameters were listed as follows: capillary voltage, 3.0 kV for ESI+ mode and 2.0 kV for ESI− mode; source temperature, 100 °C; temperature of desolvention gas, 400 °C; flow rate of cone gas, 100 L/h; flow rate of desolvention gas, 1200 L/h. The parameters of mass spectrum acquisition channels for each compound were shown in Supplementary Table S3 (the positive and negative modes were used to collect different compounds for each sample). The multiple reaction monitoring parameters for compounds are listed in Supplementary Table S3.

### *Pfkfb3* knockdown and overexpression

For *Pfkfb3* knockdown, RAW264.7 cells were transfected for 24 h with 50 nmol/L *Pfkfb3* small interfering RNAs (siRNAs) (si-*Pfkfb3*-A and si-*Pfkfb3*-B) or negative control siRNA (Genechem Co., Ltd., Shanghai, China) using Lipofectamine™ RNAiMAX (Thermo Fisher Scientific). The siRNA sequences against *Pfkfb3* are summarized in Supplementary Table S4. For *Pfkfb3* overexpression, RAW264.7 cells were transfected for 24 h with 1 μg *Pfkfb3* overexpression plasmid or empty vector (Genechem Co., Ltd.) using Lipofectamine™ 3000 (Thermo Fisher Scientific).

### Quantitative real-time PCR

TRIzol reagent was utilized to isolate total RNA, and the RevertAid First Strand cDNA Synthesis kit (Thermo Fisher Scientific) was employed to synthesize the cDNA. Quantitative real-time PCR was performed using THUNDERBIRD SYBR qPCR Mix (Toyobo Co., Ltd., Osaka, Japan) on the QuantStudio 5 Real-Time PCR System (Thermo Fisher Scientific). The relative expression of target genes was normalized to *Actb* and calculated by the 2^−ΔΔCt^ method. The primer sequences are listed in Supplementary Table S5.

### Protein quantitation and Western blot

Protein quantitation was performed by using bicinchoninic acid assay (Thermo Fisher Scientific). The denatured proteins (20 μg) were separated by 10% (*w*/*v*) SDS-PAGE electrophoresis and then transferred to nitrocellulose membranes. The membranes were incubated overnight with primary antibody at 4 °C, followed by a 1-h incubation with secondary antibody at room temperature. Protein bands were visualized by using an Odyssey 290 infrared imaging system (LI-COR Biosciences, Lincoln, NE, USA). Images were analyzed by Image J software (National Institutes of Health, Bethesda, MD, USA). β-Actin was used as a loading control.

### Statistical analysis

If data were Gaussian distributed, they are presented as mean ± SD and were analyzed by ANOVA followed by the *post hoc* Tukey-Kramer test, or by unpaired Student’s *t* test (two-tailed), as appropriate. If data were not Gaussian distributed, they are presented as median (interquartile range) and were analyzed by Kruskal–Wallis test followed by the Dunn multiple comparisons test. *P* < 0.05 was considered statistically significant. Statistical analysis was carried out using GraphPad Prism 9.0 (GraphPad Software, San Diego, CA, USA).

## Results

### SGLT2i ameliorates NAFLD in *db/db* mice

Since *db/db* mice display obesity, hyperglycemia, insulin resistance and hepatic steatosis, they are frequently used as a NAFLD model [12, 13, 27]. Our results showed that body weight, liver weight, random blood glucose, and post-load blood glucose levels during oral glucose tolerance test (OGTT) were higher in *db/db* mice than *db/m* mice (Supplementary Fig. S1a−e). A similar change was observed in serum insulin, alanine aminotransferase (ALT), aspartate aminotransferase (AST), TG, TC, low-density lipoprotein cholesterol (LDL-C), and high-density lipoprotein cholesterol (HDL-C) (Supplementary Fig. S1f−l). Compared with vehicle control, dapagliflozin and canagliflozin significantly decreased liver weight, random and post-load blood glucose levels, while remarkably increased serum insulin level in *db/db* mice (Supplementary Fig. S1a−f). Moreover, dapagliflozin significantly reduced serum ALT, TC and LDL-C levels, and canagliflozin markedly lowered serum ALT, AST, TC and LDL-C levels in *db/db* mice (Supplementary Fig. S1g−l).

**Fig. 1:**
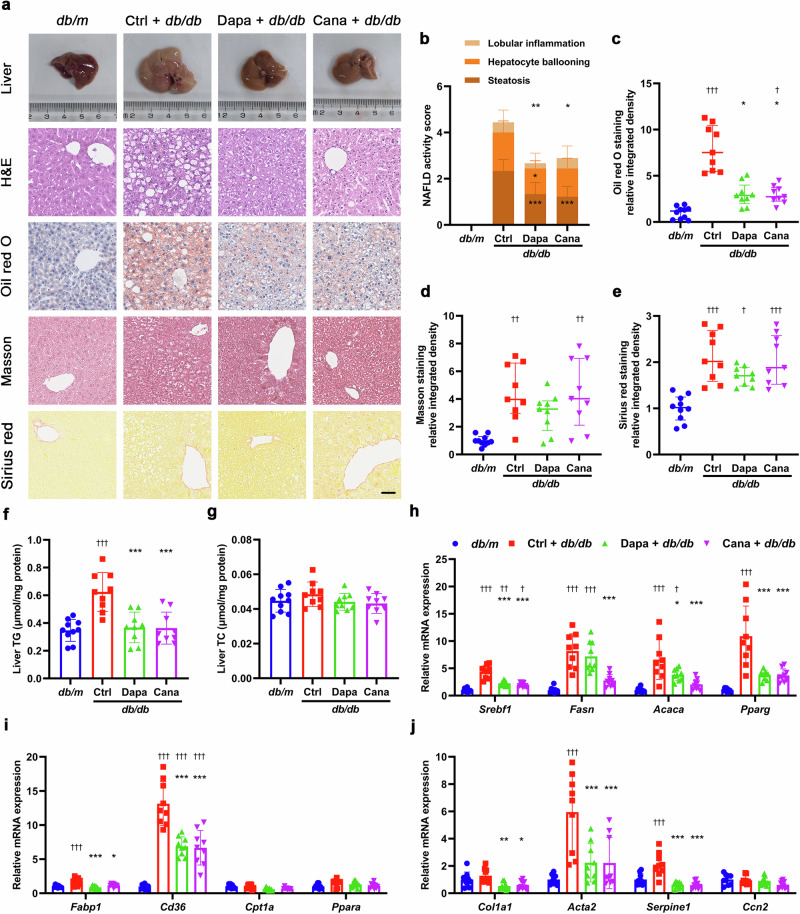
SGLT2i ameliorates NAFLD in *db/db* mice. Eight-week-old male *db/db* mice were treated for 8 weeks with dapagliflozin (1 mg·kg^−1^·d^−1^), canagliflozin (10 mg·kg^−1^·d^−1^) or vehicle. Age-matched male *db/m* mice treated with vehicle served as normal control. **a** Representative images of liver morphology, H&E staining, oil red O staining, Masson staining and Sirius red staining. Scale bar = 50 μm. **b** NAFLD activity score and individual histological scores for hepatic steatosis, hepatocellular ballooning and lobular inflammation based on H&E staining. Quantification of positive area for oil red O staining (**c**), Masson staining (**d**) and Sirius red staining (**e**). **f** Liver triglyceride (TG). **g** Liver total cholesterol (TC). Relative mRNA levels of genes related to fatty acid synthesis (**h**), fatty acid uptake and β-oxidation (**i**), and fibrosis (**j**) in liver tissues detected by quantitative real-time PCR. *n* = 9−10 per group. Data are expressed as mean ± SD or median (interquartile range). Statistical analysis was performed by ANOVA followed by the *post hoc* Tukey-Kramer test, or by Kruskal–Wallis test followed by the Dunn multiple comparisons test, as appropriate. *P < 0.05, **P < 0.01, ***P < 0.001 vs vehicle control group in *db/db* mice; ^†^*P* < 0.05, ^††^*P* < 0.01, ^†††^*P* < 0.001 vs *db/m* mice. Ctrl control, Cana canagliflozin, Dapa dapagliflozin.

Morphologically, livers of *db/db* mice were bigger than those of *db/m* mice in size and displayed yellowish color different from reddish-brown color in livers of *db/m* mice. Histological staining revealed that liver tissues of *db/db* mice were scattered with much more lipid droplets versus those of *db/m* mice. After treatment with either dapagliflozin or canagliflozin, a significant decrease in liver size and lipid droplets was observed in *db/db* mice (Fig. 1a−e). In addition, liver TG levels but not TC levels in *db/db* mice were reduced by dapagliflozin and canagliflozin (Fig. 1f, g). Dapagliflozin downregulated the mRNA levels of genes related to fatty acid synthesis (*Srebf1*, *Acaca* and *Pparg*) and uptake (*Fabp1* and *Cd36*), and fibrosis (*Col1a1*, *Acta2* and *Serpine1*). Likewise, canagliflozin downregulated the mRNA levels of genes related to fatty acid synthesis (*Srebf1*, *Fasn*, *Acaca* and *Pparg*) and uptake (*Fabp1* and *Cd36*), and fibrosis (*Col1a1*, *Acta2* and *Serpine1*) in liver tissues (Fig. 1h−j). Consistently, the protein levels of SREBF1, FASN and ACACA were decreased by dapagliflozin and canagliflozin (Supplementary Fig. S1m−p). Collectively, these findings suggested that SGLT2i ameliorated NAFLD-associated metabolic parameters and hepatic steatosis in *db/db* mice.

### SGLT2i improves NAFLD in WD-induced mouse NAFLD model

It is generally accepted that WD-fed mice represent the most translationally relevant models for studying diet-induced NASH [28, 29]. Body weight, liver weight and post-load blood glucose levels during OGTT significantly increased in WD-fed mice compared with CD-fed mice, while random blood glucose and blood glucose levels during insulin tolerance test were comparable between WD-fed and CD-fed mice (Supplementary Fig. S2a−f). Serum levels of ALT, AST, TG, TC, LDL-C and HDL-C were higher in WD-fed mice than CD-fed mice (Supplementary Fig. S2g−l). Compared with vehicle control, dapagliflozin and canagliflozin significantly decreased body weight, liver weight and post-load blood glucose levels in WD-fed mice (Supplementary Fig. S2a−f). Moreover, dapagliflozin remarkably reduced serum ALT, AST and TC levels, and canagliflozin significantly lowered serum ALT and AST levels in WD-fed mice (Supplementary Fig. S2g−l).

**Fig. 2:**
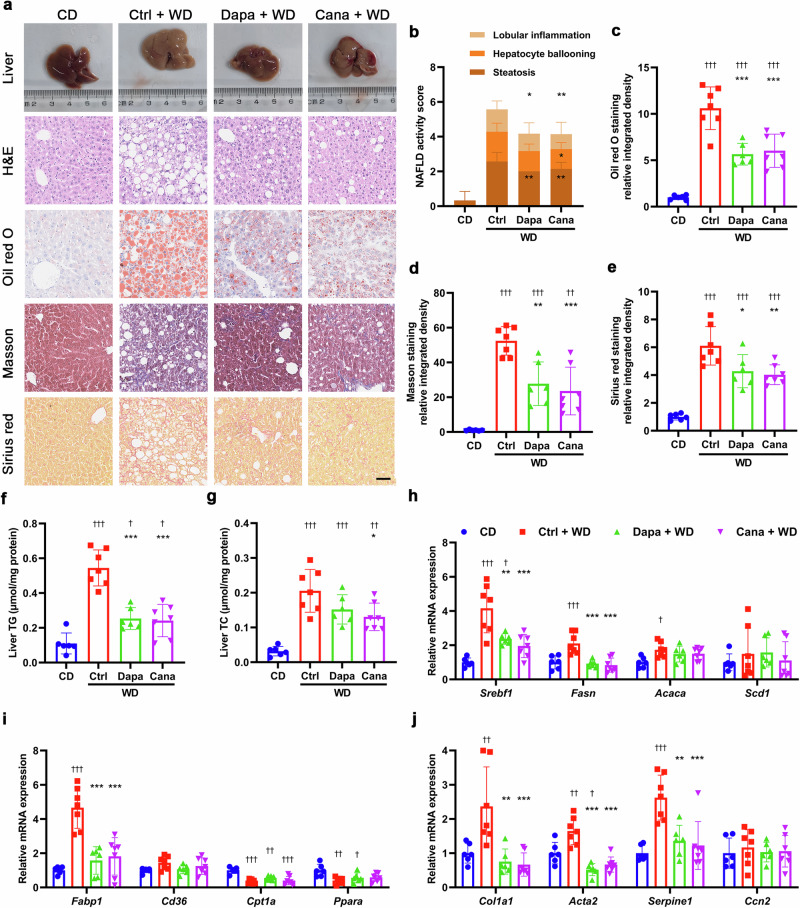
SGLT2i improves NAFLD in western diet-induced NAFLD mice. Six-week-old male C57BL/6J mice were fed on a chow diet (CD) or a western diet (WD) for 20 weeks. Subsequently, the WD-fed mice were treated for 8 weeks with dapagliflozin (1 mg·kg^−1^·d^−1^), canagliflozin (10 mg·kg^−1^·d^−1^) or vehicle. The CD-fed mice treated with vehicle were used as normal control. **a** Representative images of liver morphology, H&E staining, oil red O staining, Masson staining and Sirius red staining. Scale bar = 50 μm. **b** NAFLD activity score and individual histological scores for hepatic steatosis, hepatocellular ballooning and lobular inflammation based on H&E staining. Quantification of positive area for oil red O staining (**c**), Masson staining (**d**) and Sirius red staining (**e**). **f** Liver triglyceride (TG). **g** Liver total cholesterol (TC). Relative mRNA levels of genes related to fatty acid synthesis (**h**), fatty acid uptake and β-oxidation (**i**), and fibrosis (**j**) in the liver tissues detected by quantitative real-time PCR. *n* = 6−7 per group. Data are expressed as mean ± SD. Statistical analysis was performed by ANOVA followed by the *post hoc* Tukey-Kramer test. *P < 0.05, **P < 0.01, ***P < 0.001 vs vehicle control group in WD-fed mice; ^†^*P* < 0.05, ^††^*P* < 0.01, ^†††^*P* < 0.001 vs CD-fed mice. Ctrl control, Cana canagliflozin, Dapa dapagliflozin.

Morphologically, livers of WD-fed mice were bigger than those of CD-fed mice in size and showed yellowish color distinguished from reddish-brown color in livers of CD-fed mice. As shown by histological staining, liver tissues of WD-fed mice had more lipid droplets of varied sizes and more collagen fibers than those of CD-fed mice. Treatment with dapagliflozin or canagliflozin resulted in a notable reduction in lipid droplets and collagen fibers in this NAFLD model (Fig. 2a−e). Moreover, dapagliflozin lowered liver TG level, and canagliflozin reduced liver TG and TC levels in WD-fed mice (Fig. 2f, g). Dapagliflozin and canagliflozin downregulated the mRNA levels of genes related to fatty acid synthesis (*Srebf1* and *Fasn*) and uptake (*Fabp1*), and fibrosis (*Col1a1*, *Acta2* and *Serpine1*) in liver tissues (Fig. 2h−j). Consistently, the protein levels of SREBF1 and FASN were decreased by dapagliflozin and canagliflozin (Supplementary Fig. S2,m−p). Taken together, these results indicated that SGLT2i improved NAFLD-associated metabolic indexes, and attenuated hepatic steatosis and fibrosis in WD-induced NAFLD mice.

### SGLT2i alleviates hepatic inflammation and promotes macrophage polarization in two NAFLD models

To investigate whether SGLT2i had a direct action on hepatocytes, primary mouse hepatocytes and mouse hepatocyte cell line AML-12 cells were cultured with dapagliflozin or canagliflozin in the presence or absence of PA + OA. Evidently, dapagliflozin and canagliflozin did not have any effect on intracellular TG, TC and free cholesterol contents in primary hepatocytes (Supplementary Fig. S3a−c). Moreover, the mRNA levels of genes related to lipid metabolism were unchanged by dapagliflozin and canagliflozin (Supplementary Fig. S3d). Similarly, dapagliflozin and canagliflozin had no effects on the accumulation of lipid droplets and elevation of intracellular TG content induced by PA + OA in AML-12 cells (Supplementary Fig. S3e−h). These results suggested that the protective effect of SGLT2i on NAFLD was not due to its direct action on hepatocytes.

To clarify the localization of SGLT2 protein expression in liver tissues, immunofluorescent staining for SGLT2 was performed in liver sections of *db/db* mice and C57BL/6J mice. Results showed that SGLT2 was co-localized only with mature macrophage marker F4/80 but not hepatocyte marker albumin in liver sections (Supplementary Fig. S4a, b). Moreover, we performed immunofluorescent staining in primary mouse BMDMs, mouse peritoneal macrophages and mouse hepatocyte cell line AML-12 cells. Results showed that SGLT2 and F4/80 were co-localized in primary mouse BMDMs and peritoneal macrophages, while SGLT2 was not expressed in AML-12 cells (Supplementary Fig. S4c−e). These results suggested that macrophages but not hepatocytes might be the potential target cells of SGLT2i.

**Fig. 3:**
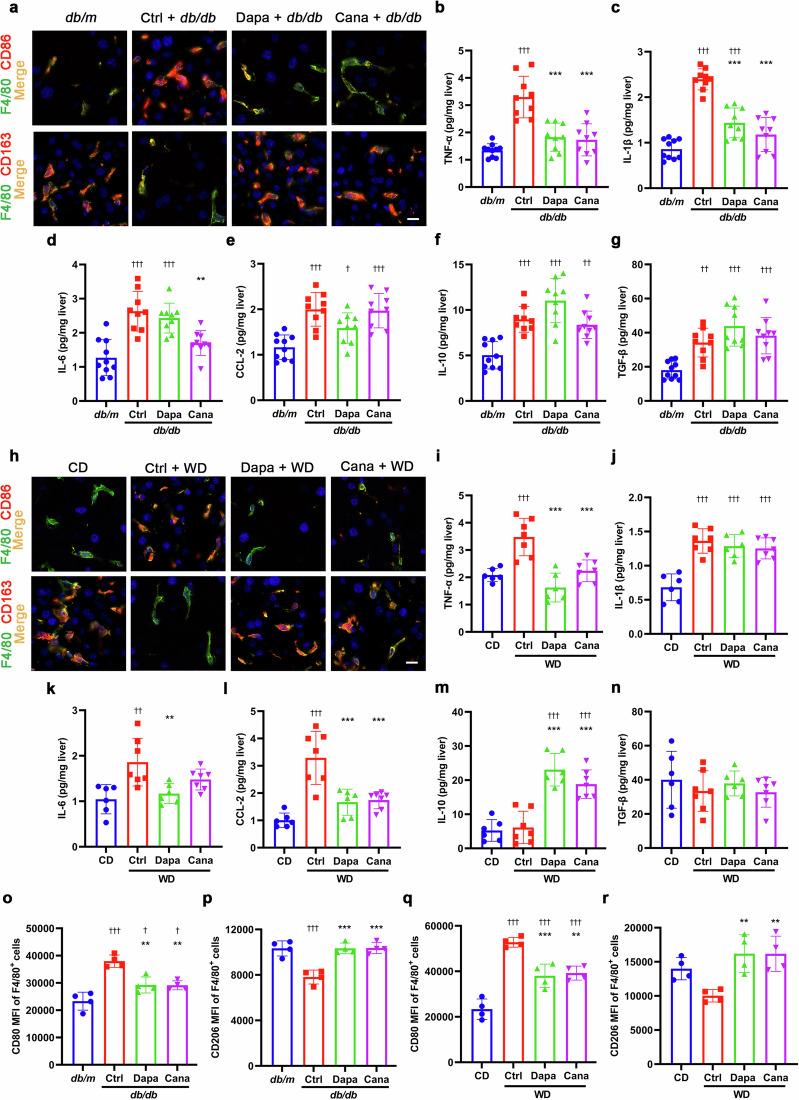
SGLT2i alleviates hepatic inflammation and improves macrophage polarization in two NAFLD models. Eight-week-old male *db/db* mice were treated for 8 weeks with dapagliflozin (1 mg·kg^−1^·d^−1^), canagliflozin (10 mg·kg^−1^·d^−1^) or vehicle. Age-matched male *db/m* mice treated with vehicle served as normal control. **a** Representative images of macrophages immunostained for F4/80 (mature macrophage marker) and CD86 (M1 marker) or CD163 (M2 marker) in liver sections. Nuclei were labeled with DAPI (blue). Scale bar = 10 μm. **b**−**g** The levels of inflammatory cytokines and chemokines in liver tissues measured by ELISA. *n* = 9−10 per group. Six-week-old male C57BL/6J mice were fed on a chow diet (CD) or a western diet (WD) for 20 weeks. Subsequently, the WD-fed mice were treated for 8 weeks with dapagliflozin (1 mg·kg^−1^·d^−1^), canagliflozin (10 mg·kg^−1^·d^−1^) or vehicle. The CD-fed mice treated with vehicle were used as normal control. **h** Representative images of macrophages immunostained for F4/80 and CD86 or CD163 in liver sections. Nuclei were labeled with DAPI (blue). Scale bar = 10 μm. **i**−**n** The levels of inflammatory cytokines and chemokines in liver tissues measured by ELISA. *n* = 6−7 per group. The mean fluorescence intensity (MFI) of M1 marker CD80 (**o**, **q**) and M2 marker CD206 (**p**, **r**) in liver macrophages isolated from *db/db* mice or WD-fed mice detected by flow cytometry. *n* = 4 per group. Data are expressed as mean ± SD. Statistical analysis was performed by ANOVA followed by the *post hoc* Tukey-Kramer test. **P < 0.01, ***P < 0.001 vs vehicle control group in *db/db* mice or WD-fed mice; ^†^*P* < 0.05, ^††^*P* < 0.01, ^†††^*P* < 0.001 vs *db/m* mice or CD-fed mice. Ctrl control, Cana canagliflozin, Dapa dapagliflozin.

Macrophages are closely linked to inflammation, and macrophage polarization plays a vital role in the development and progression of NAFLD/NASH [6, 7]. Hence, the effects of SGLT2i on macrophages and inflammation were further explored in two NAFLD models. Markedly, dapagliflozin and canagliflozin decreased CD86^+^ (M1 phenotype) cells and increased CD163^+^ (M2 phenotype) cells in liver macrophages in *db/db* mice (Fig. 3a, Supplementary Fig. S5). Furthermore, the levels of pro-inflammatory cytokines, including TNF-α and IL-1β, were significantly decreased by dapagliflozin, and the levels of TNF-α, IL-1β and IL-6 were remarkably reduced by canagliflozin in liver tissues in *db/db* mice (Fig. 3b−g). Likewise, dapagliflozin and canagliflozin decreased CD86^+^ cells and increased CD163^+^ cells in liver macrophages in WD-fed mice (Fig. 3h, Supplementary Fig. S6). In liver tissues of WD-fed mice, the levels of pro-inflammatory cytokines and chemokines TNF-α, IL-6 and CCL-2 were significantly reduced by dapagliflozin, and the levels of TNF-α and CCL-2 were markedly decreased by canagliflozin. By contrast, the level of anti-inflammatory cytokine IL-10 was increased by both dapagliflozin and canagliflozin (Fig. 3i−n).

In addition, the phenotypic marker expression in liver macrophages isolated from two NAFLD models was detected by using flow cytometry. Results showed that the mean fluorescence intensity (MFI) of CD80 (M1 phenotype) of liver macrophages was significantly decreased, while the MFI of CD206 (M2 phenotype) was markedly increased in *db/db* mice after treatment with dapagliflozin and canagliflozin (Fig. 3o, p, Supplementary Fig. S7a). Similar changes were also observed in WD-fed mice (Fig. 3q, r, Supplementary Fig. S7b). In brief, SGLT2i mitigates liver inflammation and motivates macrophage polarization from M1 to M2 phenotype.

### SGLT2i promotes macrophage polarization from M1 to M2 phenotype in mouse BMDMs and human PBMC-derived macrophages

**Fig. 4:**
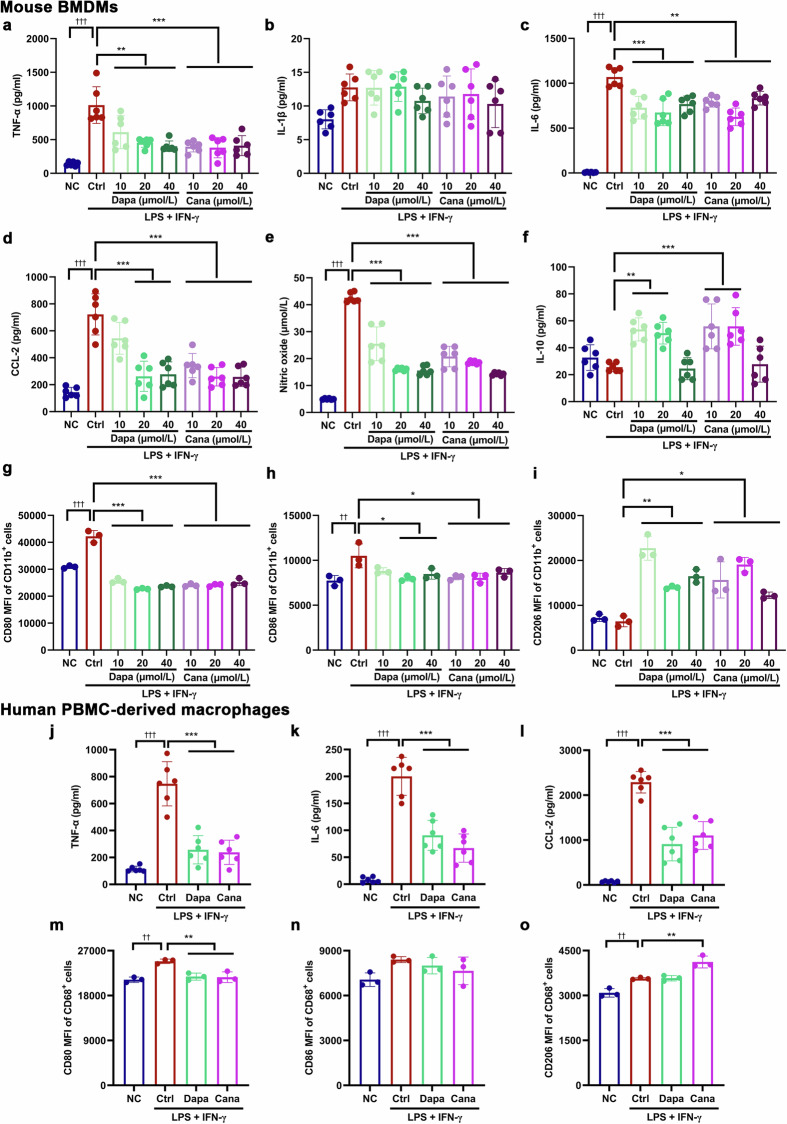
SGLT2i promotes macrophage polarization from M1 to M2 phenotype in mouse BMDMs and human PBMC-derived macrophages. Mouse bone marrow-derived macrophages (BMDMs) or human peripheral blood mononuclear cell (PBMC)-derived macrophages were incubated for 12 h with different concentrations or 20 µmol/L of dapagliflozin, canagliflozin or vehicle in the presence or absence of lipopolysaccharide (LPS, 100 ng/mL) + interferon-γ (IFN-γ, 50 ng/mL) that were used for inducing M1 polarization. The supernatant levels of pro-inflammatory (**a**−**e**) and anti-inflammatory (**f**) factors in mouse BMDMs measured by ELISA and chemical assay. *n* = 6 per group. The mean fluorescence intensity (MFI) of M1 markers CD80 and CD86 (**g**, **h**), and M2 marker CD206 (**i**) in mouse BMDMs detected by flow cytometry. *n* = 3 per group. **j**−**l** The supernatant levels of pro-inflammatory cytokines and chemokines in human PBMC-derived macrophages measured by ELISA. *n* = 6 per group. The MFI of CD80 and CD86 (**m**, **n**), and CD206 (**o**) in human PBMC-derived macrophages detected by flow cytometry. *n* = 3 per group. Data are expressed as mean ± SD. Statistical analysis was performed by ANOVA followed by the *post hoc* Tukey-Kramer test. *P < 0.05, **P < 0.01, ***P < 0.001 vs vehicle control-treated group with LPS + IFN-γ; ^††^*P* < 0.01, ^†††^*P* < 0.001 vs normal control group. Ctrl control, Cana canagliflozin, Dapa dapagliflozin, NC normal control.

Since dapagliflozin and canagliflozin alleviated hepatic inflammation and induced phenotypic transformation of macrophages in two NAFLD models, we investigated whether SGLT2i had a direct impact on macrophages. We examined the effects of dapagliflozin or canagliflozin on silent (M0) and LPS + IFN-γ-primed (M1) BMDMs. In silent BMDMs, dapagliflozin and canagliflozin significantly decreased supernatant TNF-α level, whereas increased supernatant IL-10 level (Supplementary Fig. S8a−f). In LPS + IFN-γ-primed BMDMs, dapagliflozin and canagliflozin remarkably lowered the supernatant levels of TNF-α, IL-6, CCL-2 and nitric oxide, but increased supernatant IL-10 level (Fig. 4a−f). As indicated by flow cytometry, dapagliflozin and canagliflozin did not alter CD80^+^ and CD86^+^ (M1 phenotype) cells, while led to a modest but significant elevation in CD206^+^ (M2 phenotype) cells in silent BMDMs (Supplementary Figs. S8g−i, S9a). In LPS + IFN-γ-primed BMDMs, dapagliflozin and canagliflozin markedly decreased CD80^+^ and CD86^+^ cells, but increased CD206^+^ cells (Fig. 4g−i, Supplementary Fig. S9b).

Subsequently, we investigated whether SGLT2i had similar effects on human PBMC-derived macrophages. In silent (M0) macrophages, dapagliflozin and canagliflozin did not change supernatant TNF-α, IL-6 and CCL-2 levels, and CD80^+^ and CD86^+^ cells, but led to a slight rise in CD206^+^ cells (Supplementary Figs. S8j−o, S10a). In LPS + IFN-γ-primed (M1) macrophages, dapagliflozin and canagliflozin significantly lowered the supernatant levels of TNF-α, IL-6 and CCL-2 (Fig. 4j−l). CD80^+^ cells were remarkably decreased by either dapagliflozin or canagliflozin, whereas CD206^+^ cells were significantly increased by canagliflozin (Fig. 4m−o, Supplementary Fig. S10b). These results indicated that SGLT2i had a direct anti-inflammatory action *via* promoting macrophage polarization from M1 to M2 phenotype.

### SGLT2i downregulates PFKFB3 expression in M1 macrophages

**Fig. 5:**
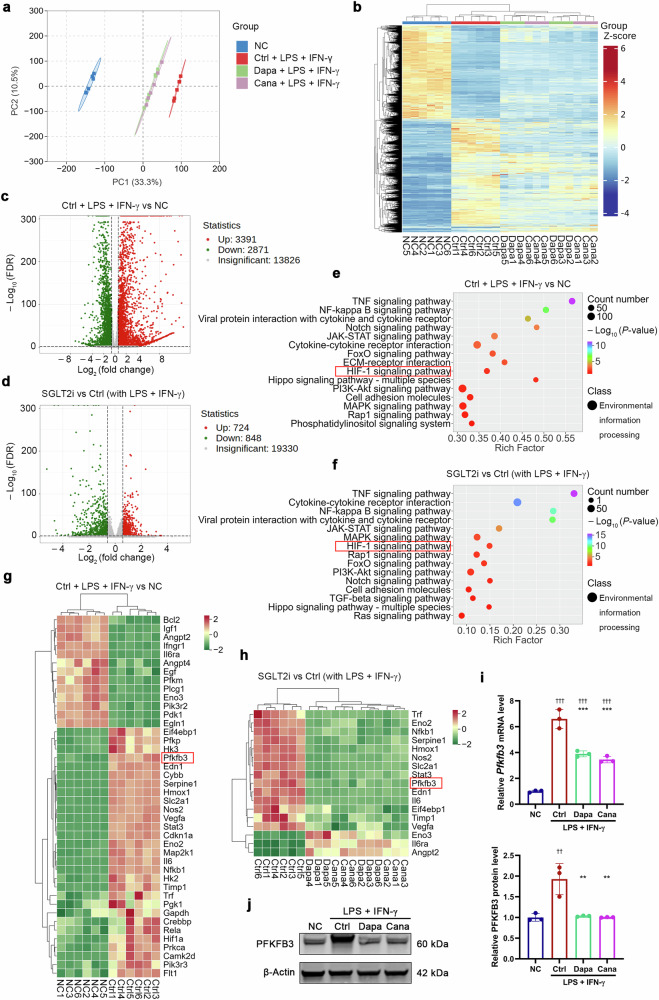
SGLT2i downregulates PFKFB3 expression in M1 macrophages. Mouse bone marrow-derived macrophages (BMDMs) were cultured for 12 h with 20 µmol/L SGLT2i (dapagliflozin or canagliflozin) or vehicle in the presence or absence of lipopolysaccharide (LPS, 100 ng/mL) + interferon-γ (IFN-γ, 50 ng/mL) that were used for inducing M1 polarization. Cells were collected for RNA sequencing (RNA-seq) analysis. **a** Principal component (PC) analysis. **b** Heatmap of the sample-to-sample differences based on RNA-seq data. **c**, **d** Volcano plot showing differential expression genes (DEGs) identified between LPS + IFN-γ and normal control groups (**c**), and between SGLT2i- and vehicle control-treated groups with LPS + IFN-γ (**d**). **e**, **f** Top 15 terms in Kyoto Encyclopedia of Genes and Genomes (KEGG) annotation involved in DEGs between LPS + IFN-γ and normal control groups (**e**), and between SGLT2i- and vehicle control-treated groups with LPS + IFN-γ (**f**). **g**, **h** Clustering heatmap of hypoxia-inducible factor-1 (HIF-1) signaling pathway target genes between LPS + IFN-γ and normal control groups (**g**), and between SGLT2i- and vehicle control-treated groups with LPS + IFN-γ (**h**). *n* = 6 per group. Genes with a *P* < 0.01 and log_2_ (fold change) <−0.585 or >0.585 were considered differentially expressed. The PFKFB3 mRNA (**i**) and protein (**j**) levels detected by quantitative real-time PCR and Western blot, respectively. *n* = 3 per group. Data are expressed as mean ± SD. Statistical analysis was performed by ANOVA followed by the *post hoc* Tukey-Kramer test. **P < 0.01, ***P < 0.001 vs vehicle control-treated group with LPS + IFN-γ; ^††^*P* < 0.01, ^†††^*P* < 0.001 vs normal control group. Ctrl control, Cana canagliflozin, Dapa dapagliflozin, NC normal control.

To investigate the underlying mechanism of macrophage phenotype shift induced by SGLT2i, we performed RNA-seq analysis in BMDMs cultured with or without dapagliflozin or canagliflozin in the presence or absence of LPS + IFN-γ that were used for inducing M1 polarization. Principal component analysis and heatmap of the sample-to-sample differences showed distinct separation between LPS + IFN-γ and normal control groups, and between SGLT2i (dapagliflozin/canagliflozin)- and vehicle control-treated groups with LPS + IFN-γ (Fig. 5a, b). We identified 6262 DEGs (3391 upregulated and 2871 downregulated) in LPS + IFN-γ group versus normal control group, and 1572 DEGs (724 upregulated and 848 downregulated) in SGLT2i-treated group versus control-treated group (Fig. 5c, d). KEGG pathway enrichment analysis showed that LPS + IFN-γ exposure elicited many DEGs associated with TNF signaling pathway, nuclear factor-κB signaling pathway, cytokine-cytokine receptor interaction, and hypoxia-inducible factor-1 (HIF-1) signaling pathway (Fig. 5e). Importantly, these pathways were also clustered when DEGs were analyzed between SGLT2i- and control-treated groups with LPS + IFN-γ (Fig. 5f).

Since upregulation of HIF-1α in macrophages induces M1 macrophage polarization [30], we focused on HIF-1 signaling pathway. Clustering analysis targeting HIF-1 pathway showed that exposure of BMDMs to LPS + IFN-γ caused 42 DEGs, including 7 upregulated genes (*Pfkp*, *Hk3*, *Pfkfb3*, *Eno2*, *Hk2*, *Pgk1* and *Gapdh*) linked to glycolysis (Fig. 5g), and that SGLT2i treatment elicited 17 DEGs, including 3 downregulated genes (*Eno2*, *Pfkfb3* and *Eno3*) related to glycolysis, in LPS + IFN-γ-primed (M1) BMDMs (Fig. 5h). PFKFB, is one of the principal regulating enzymes of glycolysis. Of the PFKFB isoenzymes, PFKFB3 has the highest activity to catalyze the synthesis of fructose-2,6-diphosphate that is the strongest allosteric activator of phosphofructokinase-1 [31]. Our quantitative real-time PCR and Western blot analyses confirmed that dapagliflozin and canagliflozin downregulated PFKFB3 mRNA and protein levels in the M1 BMDMs (Fig. 5i, j).

### SGLT2i inhibits glycolysis and promotes Kreb’s cycle in M1 macrophages

**Fig. 6:**
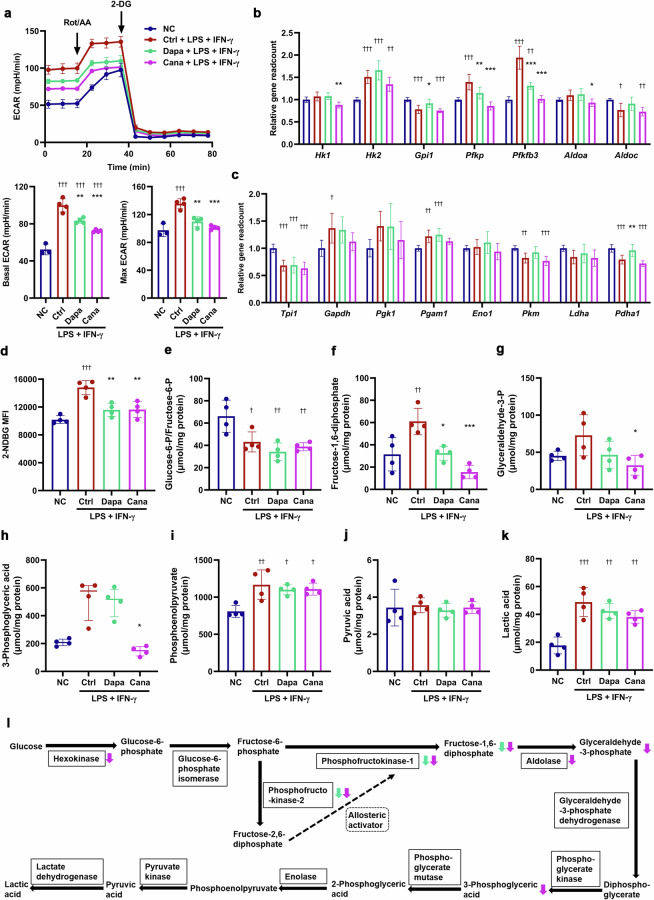
SGLT2i inhibits glycolysis in M1 macrophages. Mouse bone marrow-derived macrophages (BMDMs) were cultured for 12 h with 20 µmol/L dapagliflozin, canagliflozin or vehicle in the presence or absence of lipopolysaccharide (LPS, 100 ng/mL) + interferon-γ (IFN-γ, 50 ng/mL) that were used for inducing M1 polarization. **a** The extracellular acidification rate (ECAR), and basal and maximum ECAR capacities were determined by a respirometry and metabolomic instrument Seahorse XF96 Extracellular Flux Analyzer. 2-DG, 2-deoxy-D-glucose; Rot/AA, rotenone/antimycin A. *n* = 3−4 per group. **b**, **c** Relative readcount of genes related to glycolysis based on RNA-seq data. *n* = 6 per group. **d** Glucose uptake capacity was measured with a fluorescence-labeled D-glucose analog 2-[N-(7-nitrobenz-2-oxa-1,3-diazol-4-yl) amino]-2-deoxy-D-glucose (2-NBDG) by flow cytometry. MFI, mean fluorescence intensity. **e**−**k** The intracellular concentrations of glycolysis metabolites measured by liquid chromatography-tandem mass spectrometry (LC-MS/MS). *n* = 4 per group. **l** Schematic for the effects of SGLT2i on the key enzymes and metabolites of glycolysis in M1 macrophages. Data are expressed as mean ± SD or median (interquartile range). Statistical analysis was performed by ANOVA followed by the *post hoc* Tukey-Kramer test, or by Kruskal–Wallis test followed by the Dunn multiple comparisons test, as appropriate. *P < 0.05, **P < 0.01, ***P < 0.001 vs vehicle control-treated group with LPS + IFN-γ; ^†^*P* < 0.05, ^††^*P* < 0.01, ^†††^*P* < 0.001 vs normal control group. Ctrl control, Cana canagliflozin, Dapa dapagliflozin, NC normal control.

**Fig. 7:**
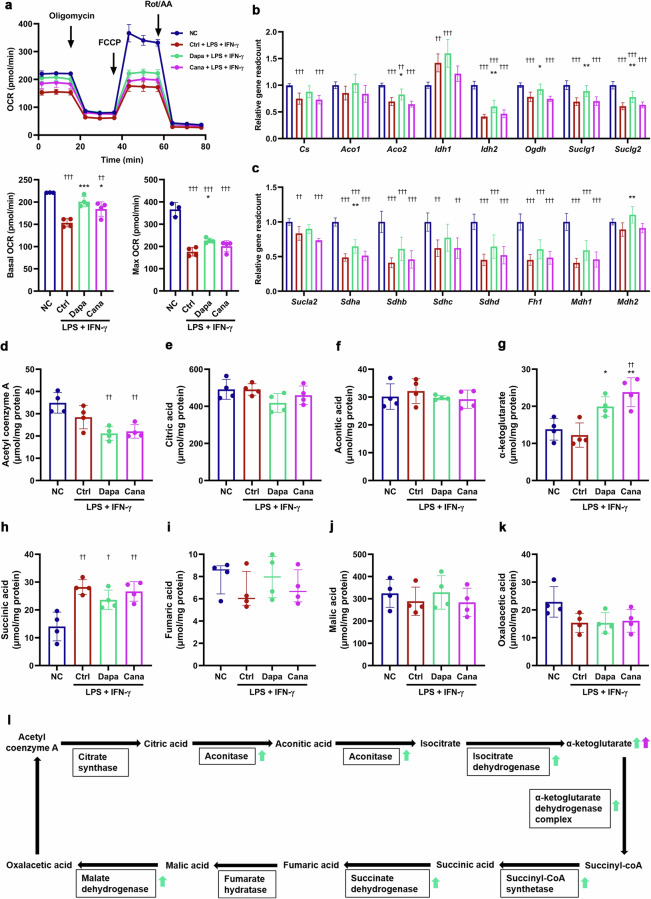
SGLT2i increases Kreb’s cycle activity in M1 macrophages. Mouse bone marrow-derived macrophages (BMDMs) were incubated for 12 h with 20 µmol/L dapagliflozin, canagliflozin or vehicle in the presence or absence of lipopolysaccharide (LPS, 100 ng/mL) + interferon-γ (IFN-γ, 50 ng/mL) that were used for inducing M1 polarization. **a** The oxygen consumption rate (OCR), and basal and maximum OCR capacities were detected by a respirometry and metabolomic instrument Seahorse XF96 Extracellular Flux Analyzer. FCCP, carbonyl cyanide 4-(trifluoromethoxy) phenylhydrazone; Rot/AA, rotenone/antimycin A. *n* = 3−4 per group. **b**, **c** Relative readcount of genes related to tricarboxylic acid (TCA) cycle activity based on RNA-seq data. *n* = 6 per group. **d**−**k** The intracellular concentrations of TCA cycle metabolites measured by liquid chromatography-tandem mass spectrometry (LC-MS/MS). *n* = 4 per group. **l** Schematic for the effects of SGLT2i on the key enzymes and metabolites of TCA cycle in M1 macrophages. Data are expressed as mean ± SD or median (interquartile range). Statistical analysis was performed by ANOVA followed by the *post hoc* Tukey-Kramer test, or by Kruskal–Wallis test followed by the Dunn multiple comparisons test, as appropriate. *P < 0.05, **P < 0.01, ***P < 0.001 vs vehicle control-treated group with LPS + IFN-γ; ^†^*P* < 0.05, ^††^*P* < 0.01, ^†††^*P* < 0.001 vs normal control group. Ctrl control, Cana canagliflozin, Dapa dapagliflozin, NC normal control.

Activation of aerobic glycolysis participates in the production of pro-inflammatory cytokines [32]. Therefore, we investigated the effects of SGLT2i on glucose metabolism in M1 macrophages. Seahorse analysis showed that dapagliflozin and canagliflozin downregulated basal and maximum ECAR capacities in LPS + IFN-γ-primed (M1) BMDMs (Fig. 6a). Conversely, dapagliflozin and canagliflozin upregulated basal OCR capacity, and dapagliflozin significantly but modestly increased maximum OCR capacity in the M1 BMDMs (Fig. 7a). Moreover, the expression of *Pfkfb3* and *Pfkp* was downregulated by dapagliflozin and canagliflozin in the M1 BMDMs (Fig. 6b, c). By contrast, the expression of multiple genes related to tricarboxylic acid (TCA) cycle (*Aco2*, *Idh2*, *Ogdh*, *Suclg1*, *Suclg2*, *Sdha* and *Mdh2*) was upregulated by dapagliflozin (Fig. 7b, c). Dapagliflozin and canagliflozin markedly inhibited glucose uptake in the M1 BMDMs (Fig. 6d). LC-MS/MS analysis showed that dapagliflozin and canagliflozin significantly decreased the intracellular concentration of fructose-1,6-diphosphate (Fig. 6e−k), and elevated the intracellular concentration of α-ketoglutarate in the M1 BMDMs (Fig. 7d−k). Consistently, dapagliflozin upregulated the expression of genes associated with fatty acid oxidation (*Acsl1*, *Acsl5*, *Cpt1a*, *Acads*, *Acadm*, *Hadh* and *Acat1*), and canagliflozin increased the expression of *Cpt1a* and *Hadh* in the M1 BMDMs (Supplementary Fig. S11). Collectively, SGLT2i inhibited glycolysis and promoted TCA cycle and mitochondrial oxidative phosphorylation in M1 macrophages (Figs. 6l and  7l), and this metabolic reprogramming contributed to macrophage polarization from M1 to M2 phenotype.

### Downregulation of PFKFB3 contributes to the anti-inflammatory action of SGLT2i in M1 macrophages

**Fig. 8:**
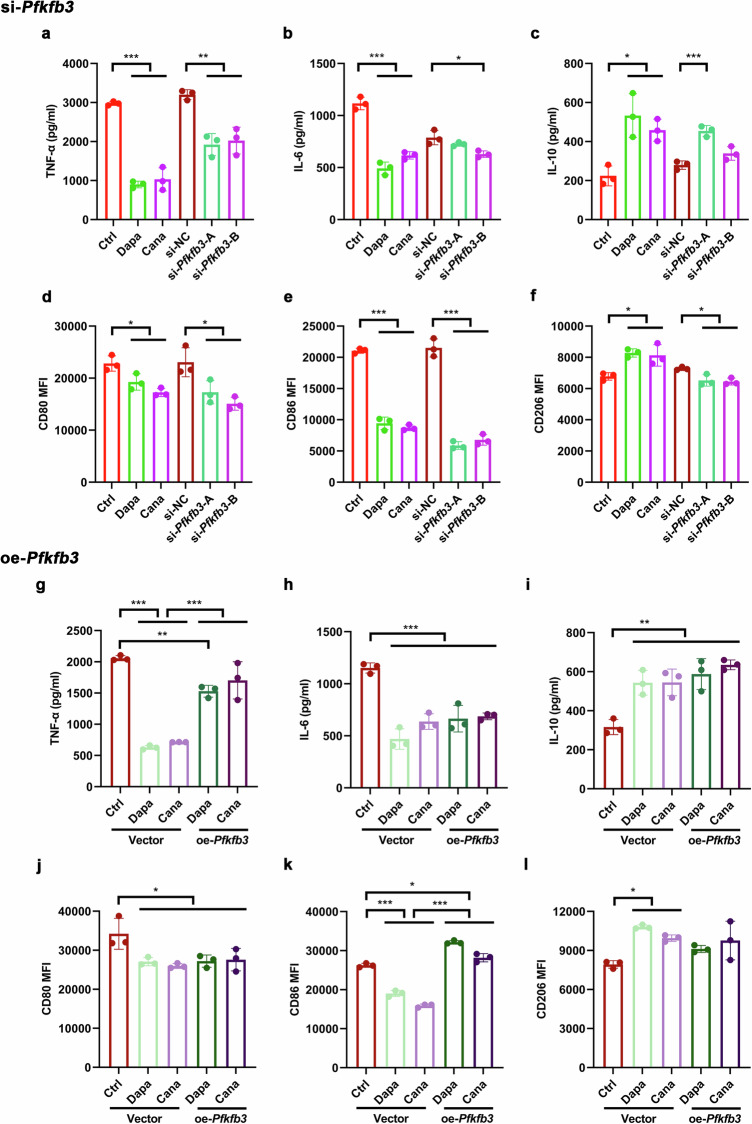
Downregulation of PFKFB3 is involved in the anti-inflammatory action of SGLT2i in M1 macrophages. The mouse macrophage cell line RAW264.7 cells were cultured for 12 h with 20 µmol/L dapagliflozin, canagliflozin or vehicle in the presence of lipopolysaccharide (LPS, 100 ng/mL) that were used for inducing M1 polarization. The cells were transfected for 24 h with small interfering RNAs (siRNAs), si-NC, si-*Pfkfb3*-A and si-*Pfkfb3*-B, and then incubated with LPS for 12 h. The supernatant levels of pro-inflammatory (**a**, **b**) and anti-inflammatory (**c**) cytokines measured by ELISA. The mean fluorescence intensity (MFI) of M1 markers CD80 and CD86 (**d**, **e**), and M2 marker CD206 (**f**) detected by flow cytometry. RAW264.7 cells were transfected with *Pfkfb3* overexpression plasmid (oe-*Pfkfb3*) or empty vector for 24 h, and then cultured with LPS and 20 µmol/L dapagliflozin, canagliflozin or vehicle for 12 h. The supernatant levels of pro-inflammatory (**g**, **h**) and anti-inflammatory (**i**) cytokines measured by ELISA. The MFI of CD80 and CD86 (**j**, **k**), and CD206 (**l**) detected by flow cytometry. *n* = 3 per group. Data are expressed as mean ± SD. Statistical analysis was performed by ANOVA followed by the *post hoc* Tukey-Kramer test. *P < 0.05, **P < 0.01, ***P < 0.001. Ctrl control, Cana canagliflozin, Dapa dapagliflozin, NC negative control.

Since glycolysis is integral to metabolic reprogramming in macrophages upon polarization [4], we explored the role of PFKFB3, a key enzyme of glycolysis, in macrophage polarization following SGLT2i treatment. Mouse macrophage cell line RAW264.7 cells were transfected with *Pfkfb3* siRNAs, si-*Pfkfb3*-A and si-*Pfkfb3*-B, for *Pfkfb3* knockdown (Supplementary Fig. S12a, b), and then were incubated with LPS to induce M1 polarization. Similar to the findings in the macrophage polarization of M1 to M2 phenotype induced by either dapagliflozin or canagliflozin (Fig. 8a−f, Supplementary Fig. S13a), we found that transfection with si-*Pfkfb3*-A significantly decreased supernatant TNF-α level, and CD80^+^ and CD86^+^ (M1 phenotype) cells, whereas it remarkably increased supernatant IL-10 level. Transfection with si-*Pfkfb3*-B exhibited an effect analogous to that with si-*Pfkfb3*-A (Fig. 8a−f, Supplementary Fig. S13a).

On the other hand, RAW264.7 cells were transfected with *Pfkfb3* overexpression plasmid or empty vector (Supplementary Fig. S12c, d), and then were incubated with dapagliflozin, canagliflozin or vehicle in the presence of LPS. Results showed that dapagliflozin and canagliflozin significantly decreased supernatant TNF-α and IL-6 levels, and CD80^+^ and CD86^+^ (M1 phenotype) cells, whereas they markedly increased supernatant IL-10 level and CD206^+^ (M2 phenotype) cells. Importantly, *Pfkfb3* overexpression reversed the effects of these SGLT2i drugs on supernatant TNF-α level and CD86^+^ cells (Fig. 8g−l, Supplementary Fig. S13b). These results indicated that downregulation of PFKFB3 was involved in the anti-inflammatory action of SGLT2i in M1 macrophages.

### Knockout of *Pfkfb3* or specific inhibition of PFKFB3 promotes macrophage polarization from M1 to M2 phenotype

**Fig. 9:**
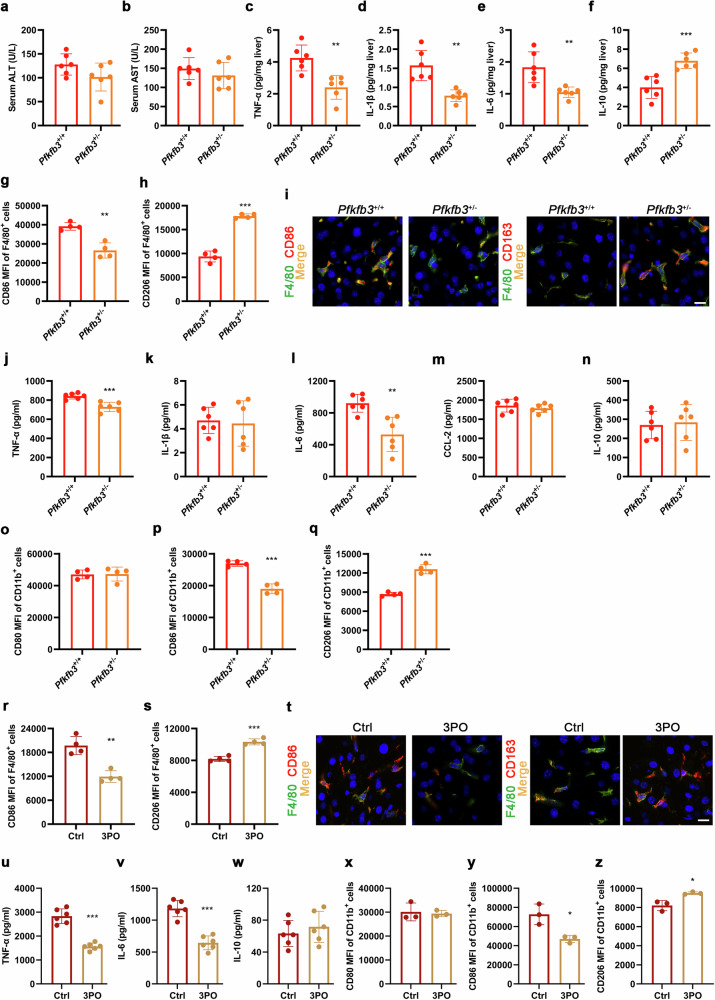
Knockout of *Pfkfb3* or specific inhibition of PFKFB3 promotes macrophage polarization from M1 to M2 phenotype. Six-week-old male global heterozygous *Pfkfb3* knockout (*Pfkfb3*^*+/−*^) mice and littermate *Pfkfb3*^*+/+*^ mice were fed on a western diet (WD) for 12 weeks. **a** Serum alanine aminotransferase (ALT). **b** Serum aspartate aminotransferase (AST). The levels of pro-inflammatory (**c**−**e**) and anti-inflammatory (**f**) cytokines in liver tissues measured by ELISA. *n* = 6 per group. The mean fluorescence intensity (MFI) of M1 marker CD86 (**g**) and M2 marker CD206 (**h**) in isolated mouse liver macrophages detected by flow cytometry. **i** Representative images of macrophages immunostained for F4/80 (mature macrophage marker) and CD86 or CD163 (another M2 marker) in liver sections. Nuclei were labeled with DAPI (blue). Scale bar = 10 μm. *n* = 4 per group. BMDMs were isolated from six-week-old male *Pfkfb3*^*+/−*^ and *Pfkfb3*^*+/+*^ mice, and were incubated for 12 h with lipopolysaccharide (LPS, 100 ng/mL) + interferon-γ (IFN-γ, 50 ng/mL) that were used for inducing M1 polarization. The supernatant levels of pro-inflammatory (**j**−**m**) and anti-inflammatory (**n**) factors measured by ELISA. *n* = 6 per group. The MFI of CD80 (another M1 marker) and CD86 (**o**, **p**), and CD206 (**q**) detected by flow cytometry. *n* = 4 per group. Six-week-old male C57BL/6J mice were fed on a WD for 8 weeks, and were treated for 4 weeks with the PFKFB3-specific inhibitor 3-(3-pyridinyl)-1-(4-pyridinyl)-2-propen-1-one (3PO; 50 mg/kg, 4 times every week) or vehicle. The MFI of CD86 (**r**) and CD206 (**s**) in isolated mouse liver macrophages detected by flow cytometry. **t** Representative images of macrophages immunostained for F4/80 and CD86 or CD163 in liver sections. Nuclei were labeled with DAPI (blue). Scale bar = 10 μm. *n* = 4 per group. BMDMs isolated from C57BL/6J mice were incubated for 12 h with 10 μmol/L 3PO or vehicle in the presence of LPS (100 ng/mL) + IFN-γ (50 ng/mL). The supernatant levels of pro-inflammatory (**u**, **v**) and anti-inflammatory (**w**) cytokines measured by ELISA. *n* = 6 per group. The MFI of CD80 and CD86 (**x**, **y**), and CD206 (**z**) detected by flow cytometry. *n* = 3 per group. Data are expressed as mean ± SD. Statistical analysis was performed by Student’s *t* test. *P < 0.05, **P < 0.01, ***P < 0.001 vs *Pfkfb3*^*+/+*^ mice or vehicle control-treated group with LPS + IFN-γ. Ctrl control.

In order to clarify the involvement of PFKFB3 in the phenotypic transformation and inflammatory response of macrophages, global heterozygous *Pfkfb3* knockout (*Pfkfb3*^*+/−*^) mice were constructed. Six-week-old male *Pfkfb3*^*+/−*^ mice and littermate *Pfkfb3*^*+/+*^ mice were fed on a WD for 12 weeks. Compared with *Pfkfb3*^*+/+*^ mice, serum ALT and AST levels of *Pfkfb3*^*+/−*^ mice showed no statistically significant difference. The levels of pro-inflammatory cytokines including TNF-α, IL-1β and IL-6 in the livers of *Pfkfb3*^*+/−*^ mice were lower, while the IL-10 level was higher than in those of *Pfkfb3*^*+/+*^ mice. In the liver macrophages of *Pfkfb3*^+/−^ mice, CD86^+^ (M1 phenotype) cells were decreased, whereas CD206^+^ and CD163^+^ (M2 phenotype) cells were increased (Fig. 9a−i, Supplementary Fig. S14a−c). In addition, BMDMs were isolated from six-week-old male *Pfkfb3*^*+/−*^ and *Pfkfb3*^*+/+*^ mice, and were incubated with LPS + IFN-γ. Results showed that the MFI of CD86, and the supernatant levels of TNF-α and IL-6 were significantly decreased, while the MFI of CD206 was remarkably increased in BMDMs isolated from *Pfkfb3*^*+/−*^ mice, when compared with those from *Pfkfb3*^*+/+*^ mice (Fig. 9j−q, Supplementary Fig. S14d).

Furthermore, six-week-old male C57BL/6J mice were fed on a WD for 8 weeks, and were treated for 4 weeks with 3PO, a PFKFB3-specific inhibitor. Results showed that CD86^+^ cells were decreased, while CD206^+^ and CD163^+^ cells were increased in the liver macrophages of mice treated with 3PO (Fig. 9r−t, Supplementary Fig. S15a−c). Moreover, BMDMs isolated from C57BL/6J mice were incubated with 3PO in the presence of LPS + IFN-γ. Results showed that 3PO significantly reduced the supernatant levels of TNF-α and IL-6, and the MFI of CD86, whereas it markedly increased the MFI of CD206 (Fig. 9u−z, Supplementary Fig. S15d). These results suggested that PFKFB3 was involved in the phenotypic transformation of macrophages both in vivo and in vitro, and specific inhibition of PFKFB3 could be used as an intervention strategy to inhibit the polarization towards M1 phenotype and/or to promote the polarization towards M2 phenotype.

### SGLT2i inhibits lipogenesis in hepatocytes *via* crosstalk with macrophages

**Fig. 10:**
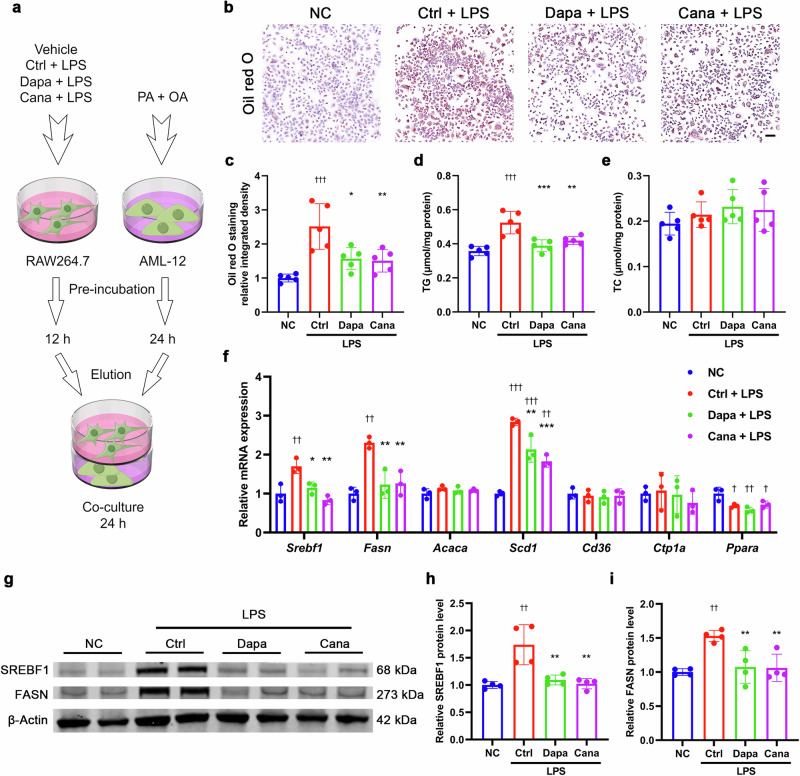
SGLT2i inhibits lipogenesis in hepatocytes *via* crosstalk with macrophages. **a** Schematic diagram of the co-culture experiments. The mouse macrophage cell line RAW264.7 cells were pre-incubated for 12 h with 20 µmol/L dapagliflozin, canagliflozin or vehicle in the presence or absence of lipopolysaccharide (LPS, 100 ng/mL), and the mouse hepatocyte cell line AML-12 cells were pre-incubated for 24 h with palmitic acid (PA, 100 μmol/L) + oil acid (OA, 200 μmol/L). Subsequently, a 24-h co-culture of RAW264.7 cells with AML-12 cells was established by using the transwell chamber. **b** Representative images of oil red O staining. Scale bar = 100 μm. **c** Quantification of positive area for oil red O staining. Intracellular triglyceride (TG) content (**d**) and total cholesterol (TC) content (**e**) measured by biochemical assay. *n* = 5 per group. **f** Relative mRNA levels of genes related to lipid metabolism detected by quantitative real-time PCR. Representative images (**g**) and quantification of the protein levels of SREBF1 (**h**) and FASN (**i**) detected by Western blot. *n* = 4 per group. Data are expressed as mean ± SD. Statistical analysis was performed by ANOVA followed by the *post hoc* Tukey-Kramer test. *P < 0.05, **P < 0.01, ***P < 0.001 vs vehicle control-treated group with LPS. ^†^*P* < 0.05, ^††^*P* < 0.01, ^†††^*P* < 0.001 vs normal control group. Ctrl control, Cana canagliflozin, Dapa dapagliflozin, NC normal control.

To explore whether SGLT2i-induced macrophage phenotype shift contributed its beneficial effect on hepatocytes, we used co-culture of macrophages with hepatocytes (Fig. 10a). As shown by oil red O staining, co-culture with LPS-primed RAW264.7 cells (mouse macrophage cell line) significantly accelerated lipid droplet accumulation induced by PA + OA in AML-12 cells (mouse hepatocyte cell line), while treatment with dapagliflozin or canagliflozin in LPS-primed RAW264.7 cells remarkably attenuated this effect (Fig. 10b, c). These observations were supported by a similar alteration in intracellular TG content, albeit intracellular TC content remained unchanged in AML-12 cells (Fig. 10d, e). Consistently, co-culture with LPS-primed RAW264.7 cells upregulated the mRNA levels of genes related to lipogenesis (*Srebf1*, *Fasn* and *Scd1*) and the protein levels of SREBF1 and FASN in AML-12 cells pre-incubated with PA + OA, while exposure of LPS-primed RAW264.7 cells to dapagliflozin or canagliflozin significantly diminished these effects (Fig. 10f−i). These results indicated that SGLT2i inhibited lipogenesis in hepatocytes *via* crosstalk with macrophages.

## Discussion

Evidence from multiple clinical trials and basic studies shows that SGLT2i not only improves glycemic control but also exhibits encouraging advantages for NAFLD treatment [8–10], albeit the precise mechanism remains uncertain. In this study, we demonstrated that dapagliflozin and canagliflozin ameliorated NAFLD-associated metabolic indexes, hepatic steatosis, inflammation and fibrosis in both *db/db* mice and WD-induced NAFLD mice. Moreover, these SGLT2i drugs decreased M1 macrophages and increased M2 macrophages in liver tissues in these two models. Consistently, we showed that SGLT2i had a direct effect on macrophage polarization from M1 to M2 phenotype in vitro. Notably, dapagliflozin and canagliflozin suppressed glycolysis by downregulating PFKFB3, but promoted TCA cycle and fatty acid β-oxidation, suggestive of a metabolic reprogramming in macrophages. As a result, dapagliflozin and canagliflozin polarized M1 phenotype into M2 phenotype, alleviated inflammatory response, and protected hepatocytes. These findings indicated that SGLT2i might be a promising drug for treating NAFLD and that macrophages and PFKFB3 would be the potential targets for NAFLD treatment.

Our study showed that dapagliflozin and canagliflozin exhibited a remarkable benefit in improving hepatic lipid metabolism and mitigating liver damage in two NAFLD models, which aligns with previous animal studies [33, 34]. Consistently, in patients with type 2 diabetes and NAFLD, SGLT2i decreased liver fat content and lowered serum liver enzyme level [9, 35]. Moreover, SGLT2i had the potential to hinder or mitigate liver fibrosis [36]. Nevertheless, the complete understanding of these fundamental processes needed further investigation. In our study, SGLT2 was expressed in macrophages rather than hepatocytes in liver tissues of *db/db* mice as evaluated by immunofluorescence. Other reports also showed that SGLT2 expression could be observed in macrophages, T cells, and stellate cells in mouse livers [34, 36]. These results suggested that SGLT2i might have a direct impact on liver macrophages, in addition to an indirect action secondary to its glucose-lowering effect.

Liver contains the highest percentage of macrophages compared with other solid organs in the body [37]. NASH, a critical phase of NAFLD, is strongly linked to activation of macrophages [38, 39]. The disease severity is exacerbated by shifting liver macrophages towards a pro-inflammatory phenotype (M1) [40]. Therefore, approaches to limit M1 polarization and/or promote M2 activation could potentially safeguard against heightened inflammation, thereby mitigating NASH progression. We found that dapagliflozin and canagliflozin downregulated the expression of M1 phenotype biomarkers and upregulated the expression of M2 phenotype biomarkers, thereby leading to inflammation resolution both in vivo and in vitro. Notably, co-culture of dapagliflozin- or canagliflozin-treated LPS-activated M1 macrophages with hepatocytes could alleviate the LPS-activated M1 macrophage-induced lipogenesis in hepatocytes, while mono-culture of hepatocytes with dapagliflozin or canagliflozin did not show such a beneficial effect. These results suggested that the benefit of SGLT2i on liver was secondary to its action in macrophages. Consistently, another SGLT2i empagliflozin facilitates adipose utilization and browning, alleviates inflammation, and enhanced insulin sensitivity through polarizing M2 macrophages in obese mice [11]. These observations support the concept that macrophages would be a promising target for NAFLD treatment.

Macrophages exhibit unique metabolic traits that are associated with the functional condition, that is commonly referred to as metabolic reprogramming [4, 41]. In general, pro-inflammatory macrophages (M1) primarily depend on glycolysis and show dysfunction in TCA cycle and mitochondrial oxidative phosphorylation, while anti-inflammatory macrophages (M2) rely more on mitochondrial oxidative phosphorylation [42, 43]. Complementally, glycolysis increases pro-inflammatory cytokine production and promotes macrophage phagocytosis, enhancing innate immunity [44]. M1 macrophages also exhibit an interrupted TCA cycle. As a result of the first break at isocitrate dehydrogenase, citrate accumulates. Citrate is utilized for prostaglandin production, fatty acid synthesis and membrane biosynthesis [43, 45]. The second break occurs at succinate dehydrogenase, leading to succinate accumulation. Succinate is an inflammatory signal that stabilizes HIF-1α, thereby promoting IL-1β production [46]. Therefore, the disturbed TCA cycle further reinforces the inflammatory phenotype.

Our study demonstrated that dapagliflozin and canagliflozin inhibited glycolysis and activated mitochondrial oxidative phosphorylation and that they upregulated the expression of multiple genes related to TCA cycle and the level of α-ketoglutarate in M1 macrophages. It is worth noting that α-ketoglutarate plays an important signaling role for eliciting anti-inflammatory gene expression program *via* epigenetic modifications, and suppressing the classical activation program synchronously [47]. These findings might explain why SGLT2i polarized macrophages from M1 to M2 phenotype. We also noticed the difference between dapagliflozin and canagliflozin in modulating intracellular metabolic reprogramming. There might be the following reasons. First, despite being the same class of SGLT2i, dapagliflozin and canagliflozin are not exactly the same drug. Dapagliflozin inhibits SGLT2, while canagliflozin inhibits both SGLT2 and SGLT1 [48] which may lead to more enzymes and more metabolites of glycolysis affected by canagliflozin than dapagliflozin. Second, a previous study revealed a double mode of action for canagliflozin, but not for dapagliflozin. Canagliflozin inhibited the glutamate dehydrogenase and mitochondrial electron transport chain complex I at clinically relevant concentrations. This dual inhibition specifically prevented replenishment of TCA cycle metabolites by glutamine (anaplerosis) [49]. These results might partly explain why canagliflozin had less impact on TCA cycle than dapagliflozin.

How SGLT2i affects glycolysis and polarized macrophages is another important question. By using RNA-seq analysis, we identified HIF-1 signaling pathway as a potential mediator. HIF is a transcription factor that plays an important role in macrophage-mediated immune responses, and masterminds a shift to cell type-specific transcriptional outputs in cellular metabolism [30]. Macrophage activation induces both aerobic glycolysis and HIF-1α stabilization, and they reinforce one another in a positive feedback loop that further enhances the macrophage activation [50] and pro-inflammatory cytokine production [32]. In addition, HIF-1α is required for the transcriptional regulation of many glycolytic genes, including those encoding glucose transporter 1, lactate dehydrogenase A, hexokinase, phosphofructokinase, pyruvate kinase and glyceraldehyde-3-phosphate dehydrogenase [51]. As one of the key enzymes in glycolysis, pyruvate kinase M2 has a key role in macrophage polarization, and lactylation of this enzyme suppresses inflammatory metabolic adaptation in M1 macrophages [52]. Therefore, controlling intracellular glucose metabolism in macrophages is a crucial approach to limit macrophage activation. Our results showed that either dapagliflozin or canagliflozin reduced the expression level of PFKFB3, a key enzyme of glycolysis, in M1 macrophages. It has been documented that *Pfkfb3* overexpression can elevate the level of fructose-2,6-diphosphate, which is the strongest allosteric activator of phosphofructokinase-1, resulting in enhanced glycolytic flux and reinforced pro-inflammatory function [31, 53]. By using genetic manipulation and pharmacological inhibition, we proved that PFKFB3 participated in modulating the phenotypic transformation of macrophages both in vivo and in vitro, and PFKFB3 was involved in the SGLT2i-induced macrophage phenotype shift. These results suggested that specific inhibition of PFKFB3 by SGLT2i or other agents might be a promising strategy for modulating macrophage polarization and treating NAFLD.

## Conclusion

We demonstrate that SGLT2i ameliorates hepatic steatosis, inflammation and fibrosis in two NAFLD models. The protective effects of SGLT2i are achieved by inducing macrophage polarization from M1 to M2 phenotype, rather than by a direct action on hepatocytes. Importantly, SGLT2i suppresses glycolysis by downregulating PFKFB3, thereby triggering intracellular metabolic reprogramming and inducing phenotypic transformation of macrophages, which is one of the important mechanisms of SGLT2i in improving NAFLD. These observations broaden our understanding for the pharmacological actions of SGLT2i and provide a novel insight into the development of SGLT2i or other PFKFB3 inhibitors for NAFLD treatment.

## Supplementary information

Supplementary Information
