# Supplementary material for: SGLT2 inhibitors ameliorate NAFLD in mice via downregulating PFKFB3, suppressing glycolysis and modulating macrophage polarization
Source: Acta Pharmacol Sin. 2024 Sep 18;45(12):2579–97. doi: 10.1038/s41401-024-01389-3 (PMC11579449; doi:10.1038/s41401-024-01389-3)
Supplement: Supplementary file 1 — Supplementary Information [file 41401_2024_1389_MOESM1_ESM.docx]

*Supplementary Information*

SGLT2 inhibitors ameliorate NAFLD in mice *via* downregulating PFKFB3, suppressing glycolysis and modulating macrophage polarization

**Contents in Supplementary File**

This supplementary file includes supplementary methods, 15 supplementary figures and 5 supplementary tables.

**SUPPLEMENTARY METHODS**

**Glucose monitoring, oral glucose tolerance test (OGTT) and insulin tolerance test (ITT)**

Mouse peripheral blood was sampled from tail vein, and glucose levels were measured using a portable OneTouch Ultra glucometer (LifeScan, Milpitas, CA, USA) with glucose oxidase technique. If the glucose concentration exceeded 33.3 mmol/L (the upper detection limit of the glucometer), it was recorded as 33.3 mmol/L.

Random blood glucose was monitored weekly at 9:00 a.m.. For OGTT, after fasting for 12 h, mice were intragastrically administered with 1.0 or 2.0 g/kg glucose, and blood glucose was assessed at baseline, and at 30, 60 and 120 min after the glucose loading. For ITT, mice were fasted for 6 h and then intraperitoneally injected with 1.0 U/kg insulin. Blood glucose was measured at baseline, and at 15, 30, 60 and 120 min after the insulin injection.

**Serum biochemical analysis and insulin assay**

Blood was taken from mouse inner canthus before sacrifice. Serum samples were prepared by centrifuging whole blood at 2000 × *g* for 10 min. Subsequently, supernatant was collected and stored at −80 ℃ for the measurement. Serum alanine aminotransferase (ALT), aspartate aminotransferase (AST), triglyceride (TG), total cholesterol (TC), low-density lipoprotein cholesterol (LDL-C) and high-density lipoprotein cholesterol (HDL-C) were measured with Automatic Biochemical Analyzer (BS-350E; Mindray, Shenzhen, China) according to the manufacturer's instructions. Serum insulin was measured using specific ELISA kit (Alpco, Salem, NH, USA) following the manufacturer's protocols. The kits are summarized in Table S1.

**Cell lipid analysis**

Cell samples were lysed in radioimmunoprecipitation assay lysis buffer (Applygen, Beijing, China). Biochemical assay kits for TG, TC and free cholesterol (all from Applygen) were used to detect the lipid profile in cell lysates according to the manufacturers' protocols. The kits are listed in Table S1.

**Isolation and culture of primary mouse hepatocytes**

Eight-week-old male C57BL/6J mice were perfused with collagenase through the portal vein to isolate primary hepatocytes as described previously ^[1, 2]^. By flushing with RPMI-1640 medium, the perfused liver was passed through a 100-μm screening size filter. Hepatocytes were centrifuged at 50 × *g* for 3 min, and were resuspended in RPMI-1640 medium with 10% fetal bovine serum (FBS) and 1% penicillin-streptomycin. Hepatocytes were plated in 6-well dishes coated with rat collagen type I (Sigma-Aldrich, St. Louis, MO, USA) for further experiments. After a 24-h pre-incubation, primary hepatocytes were cultured for 24 h with 20 μmol/L dapagliflozin, canagliflozin or vehicle in the presence or absence of palmitic acid (PA; 100 μmol/L) + oil acid (OA; 200 μmol/L) that were used for inducing an in vitro model of liver lipid accumulation.

**Isolation and culture of primary mouse liver macrophages**

The isolation of primary liver macrophages was based on perfusion technique as previously described ^[1, 2]^. Originally, liver macrophages were from non-parenchymal cells of collagenase-perfused mouse liver ^[3-5]^. Briefly, total liver cells were obtained after perfusion and non-parenchymal cells were separated by low-speed centrifugation (50 × *g*) for 3 min. Hepatocytes were in the pellet. The supernatant was centrifuged at 500 × *g* for 5 min to collect non-parenchymal cells. The resuspended non-parenchymal cells were added to a density gradient of 20%/50% Percoll (Sigma-Aldrich) followed by a centrifugation at 800 × *g* for 15 min at 4 ℃. Macrophages were in the interphase layer of 20% and 50% Percoll. After washing, the purified macrophages were cultured in RPMI-1640 medium with 10% FBS and 1% penicillin-streptomycin.

**Isolation and culture of primary mouse peritoneal macrophages**

Isolation of mouse peritoneal macrophages was performed as described previously ^[6]^. Eight-week-old male C57BL/6J mice were used for peritoneal lavage with 10 mL cold phosphate buffered saline (PBS). The lavage fluid was collected and centrifuged at 100 × *g* for 5 min. Cells were obtained and resuspended in RPMI-1640 medium with 10% FBS and 1% penicillin-streptomycin. Cells were seeded in 6-well dishes at a density of 5 × 10^6^ cell/mL. After a 4-h pre-incubation, the non-adherent cells were removed by gently washing with PBS. Subsequently, the adherent cells were cultured for further experiments.

**REFERENCES**

1. Liu J, Yang K, Yang J, Xiao W, Le Y, Yu F*,* et al. Liver-derived fibroblast growth factor 21 mediates effects of glucagon-like peptide-1 in attenuating hepatic glucose output. EBioMedicine. 2019;41:73-84.

2. Cui X, Feng J, Wei T, Zhang L, Lang S, Yang K*,* et al. Pancreatic alpha cell glucagon-liver FGF21 axis regulates beta cell regeneration in a mouse model of type 2 diabetes. Diabetologia. 2023;66:535-50.

3. Huang S, Wu Y, Zhao Z, Wu B, Sun K, Wang H*,* et al. A new mechanism of obeticholic acid on NASH treatment by inhibiting NLRP3 inflammasome activation in macrophage. Metabolism. 2021;120:154797.

4. Li W, Yang Y, Yang L, Chang N, Li L. Monocyte-derived Kupffer cells dominate in the Kupffer cell pool during liver injury. Cell Rep. 2023;42:113164.

5. Zhang Q, Wei J, Liu Z, Huang X, Sun M, Lai W*,* et al. STING signaling sensing of DRP1-dependent mtDNA release in kupffer cells contributes to lipopolysaccharide-induced liver injury in mice. Redox Biol. 2022;54:102367.

6. Jeljeli M, Riccio LGC, Chouzenoux S, Moresi F, Toullec L, Doridot L*,* et al. Macrophage immune memory controls endometriosis in mice and humans. Cell Rep. 2020;33:108325.

**SUPPLEMENTARY FIGURES**

**
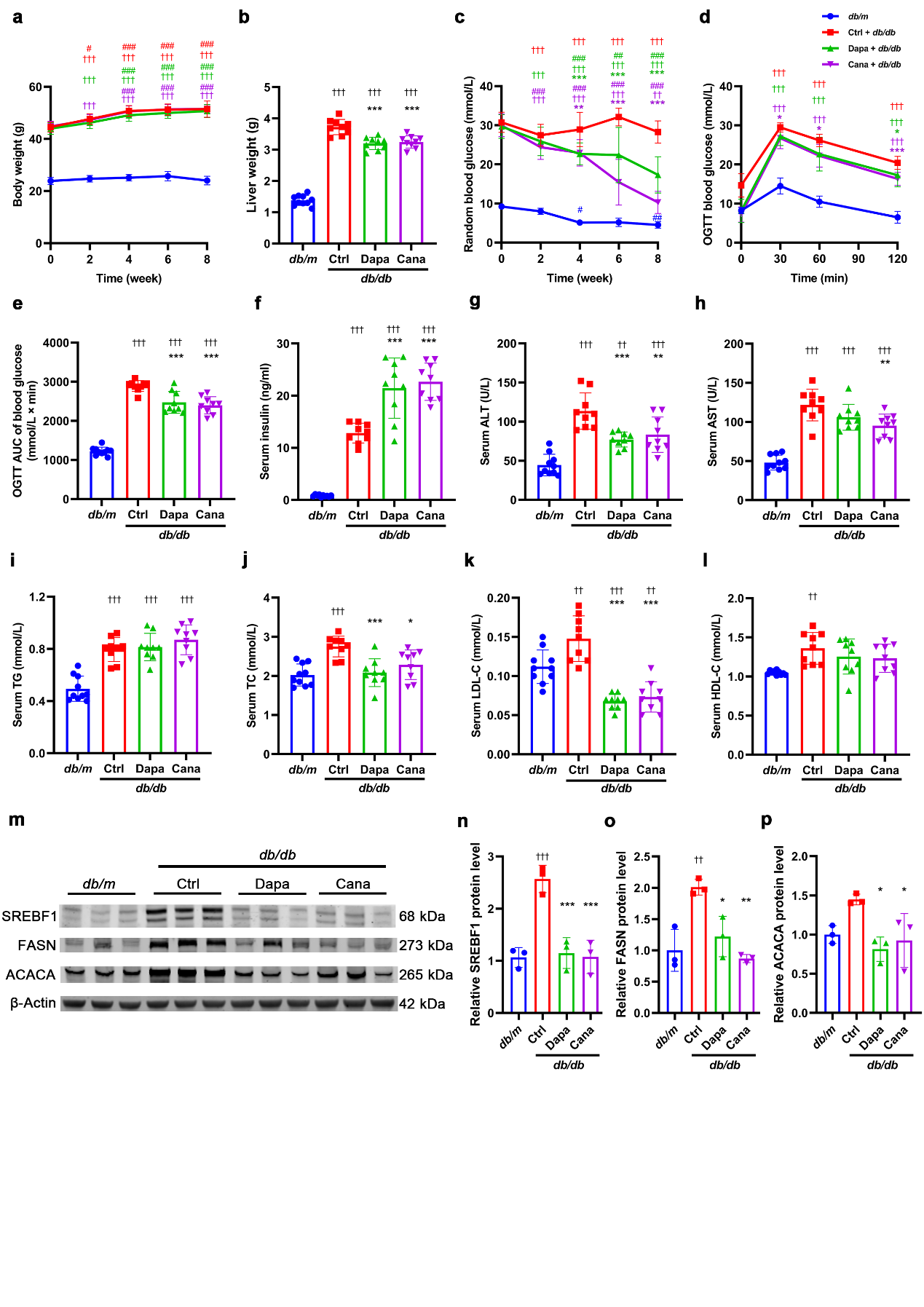
**

**Fig. S1 SGLT2i ameliorates NAFLD-related metabolic parameters in *db/db* mice.**

Eight-week-old male *db/db* mice were treated for 8 weeks with dapagliflozin (1 mg·kg^-1^·d^-1^), canagliflozin (10 mg·kg^-1^·d^-1^) or vehicle. Age-matched male *db/m* mice treated with vehicle were used as normal control. **a** Body weight. **b** Liver weight. **c** Random blood glucose. **d** Blood glucose during the oral glucose tolerance test (OGTT). **e** The area under curve (AUC) for blood glucose during the OGTT. **f** Serum insulin. **g** Serum alanine aminotransferase (ALT). **h** Serum aspartate aminotransferase (AST). **i** Serum triglyceride (TG). **j** Serum total cholesterol (TC). **k** Serum low-density lipoprotein cholesterol (LDL-C). **l** Serum high-density lipoprotein cholesterol (HDL-C). *n* = 9−10 per group. **m−p** Representative images (**m**) and quantification of the protein levels of SREBF1 (**n**), FASN **(o**) and ACACA (**p**) detected by Western blot. *n* = 3 per group. Data are expressed as mean ± SD. Statistical analysis was performed by ANOVA followed by the post hoc Tukey-Kramer test. **P*<0.05, ***P*<0.01, ****P*<0.001 vs vehicle control group in *db/db* mice; ^††^*P*<0.01, ^†††^*P*<0.001 vs *db/m* mice; ^#^*P*<0.05, ^##^*P*<0.01, ^###^*P*<0.001, post-treatment vs pre-treatment in the same group. Ctrl, control; Cana, canagliflozin; Dapa, dapagliflozin.

**
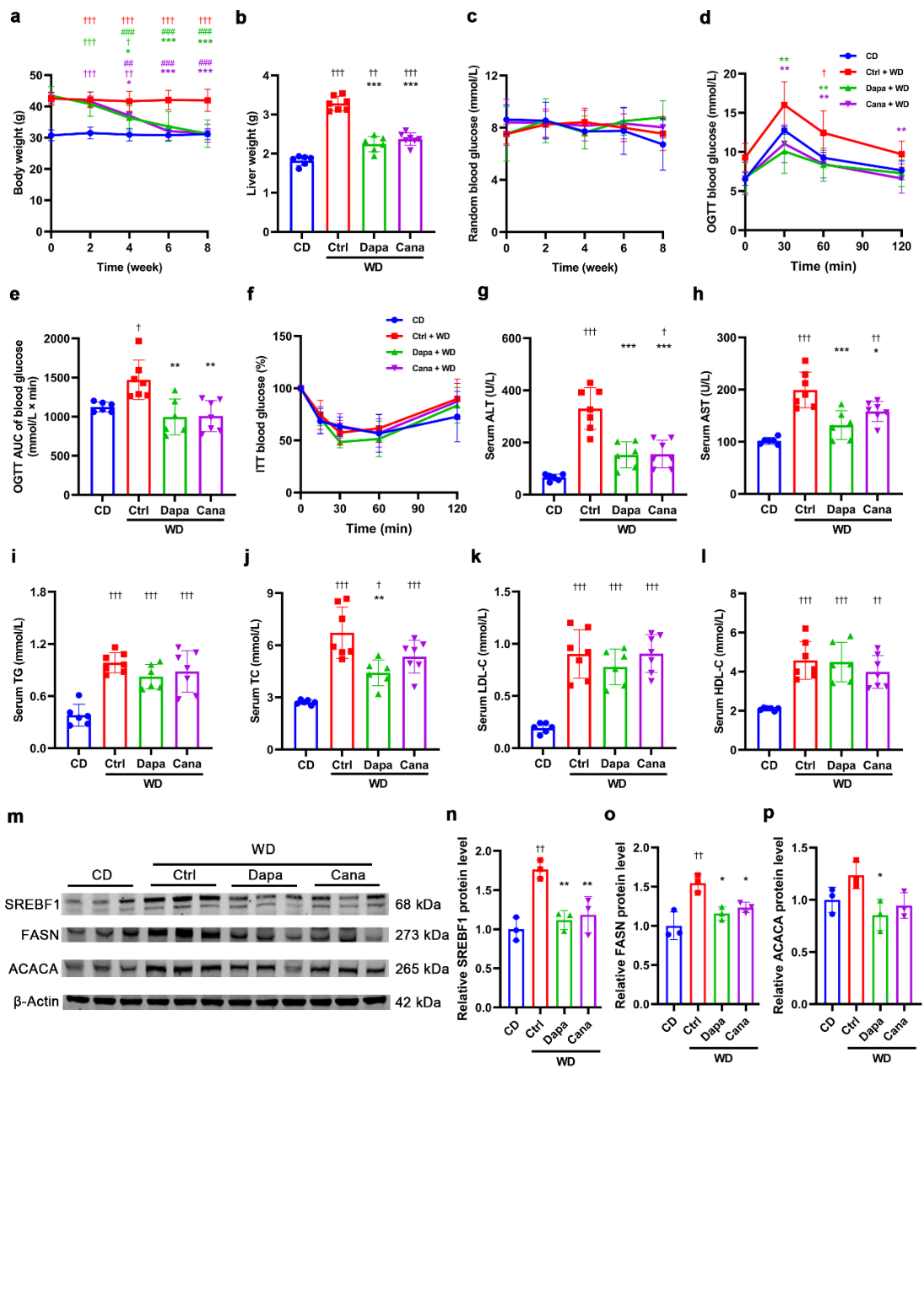
**

**Fig. S2 SGLT2i improves NAFLD-related metabolic parameters in western diet-induced NAFLD mice.**

Six-week-old male C57BL/6J mice were fed on a chow diet (CD) or a western diet (WD) for 20 weeks. Subsequently, the WD-fed mice were treated for 8 weeks with dapagliflozin (1 mg·kg^-1^·d^-1^), canagliflozin (10 mg·kg^-1^·d^-1^) or vehicle. The CD-fed mice treated with vehicle were included as normal control. **a** Body weight. **b** Liver weight. **c** Random blood glucose. **d** Blood glucose during the oral glucose tolerance test (OGTT). **e** The area under curve (AUC) for blood glucose during the OGTT. **f** Blood glucose during the insulin tolerance test (ITT). **g** Serum alanine aminotransferase (ALT). **h** Serum aspartate aminotransferase (AST). **i** Serum triglyceride (TG). **j** Serum total cholesterol (TC). **k** Serum low-density lipoprotein cholesterol (LDL-C). **l** Serum high-density lipoprotein cholesterol (HDL-C). *n* = 6−7 per group. **m−p** Representative images (**m**) and quantification of the protein levels of SREBF1 (**n**), FASN (**o**) and ACACA (**p**) detected by Western blot. *n* = 3 per group. Data are expressed as mean ± SD. Statistical analysis was performed by ANOVA followed by the post hoc Tukey-Kramer test. **P*<0.05, ***P*<0.01, ****P*<0.001 vs vehicle control group in WD-fed mice; ^†^*P*<0.05, ^††^*P*<0.01, ^†††^*P*<0.001 vs CD-fed mice; ^##^*P*<0.01, ^###^*P*<0.001, post-treatment vs pre-treatment in the same group. Ctrl, control; Cana, canagliflozin; Dapa, dapagliflozin.

**
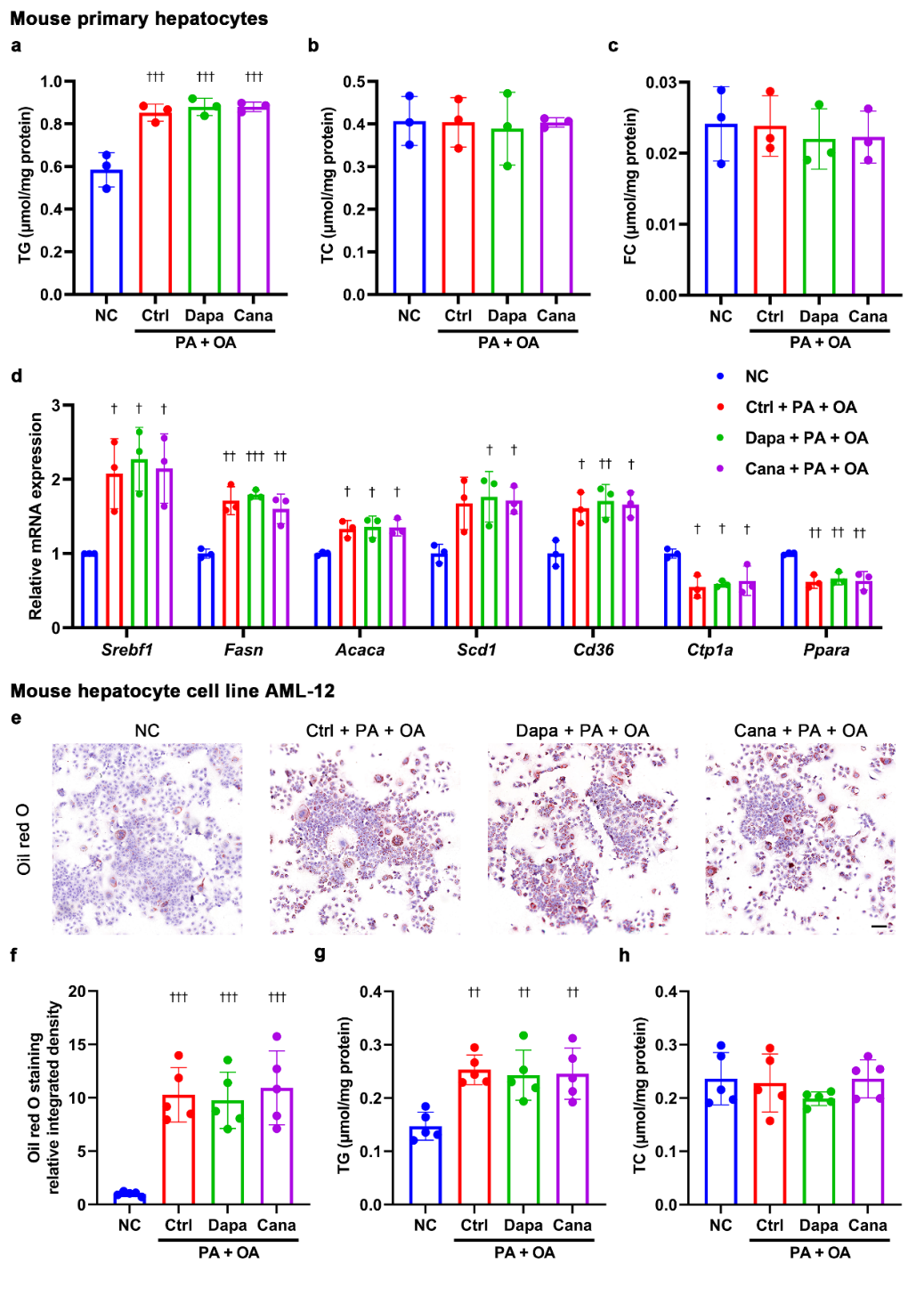
**

**Fig. S3 SGLT2i has no direct effect on hepatocytes.**

Primary mouse hepatocytes were cultured for 24 h with 20 µmol/L dapagliflozin, canagliflozin or vehicle in the presence or absence of palmitic acid (PA, 100 μmol/L) + oil acid (OA, 200 μmol/L). **a−c** Intracellular triglyceride (TG) (**a**), total cholesterol (TC) (**b**) and free cholesterol (FC) (**c**) contents measured by biochemical assay. **d** Relative mRNA levels of genes related to lipid metabolism detected by quantitative real-time PCR. *n* = 3 per group. Mouse hepatocyte cell line AML-12 cells were cultured for 24 h with 20 µmol/L dapagliflozin, canagliflozin or vehicle in the presence or absence of PA (100 μmol/L) + OA (200 μmol/L). **e** Representative images of oil red O staining. Scale bar = 100 μm. **f** Quantification of positive area for oil red O staining. **g, h** Intracellular TG (**g**) and TC (**h**) contents measured by biochemical assay. *n* = 5 per group. Data are expressed as mean ± SD. Statistical analysis was performed by ANOVA followed by the post hoc Tukey-Kramer test. ^†^*P*<0.05, ^††^*P*<0.01, ^†††^*P*<0.001 vs normal control group. Ctrl, control; Cana, canagliflozin; Dapa, dapagliflozin; NC, normal control.

**
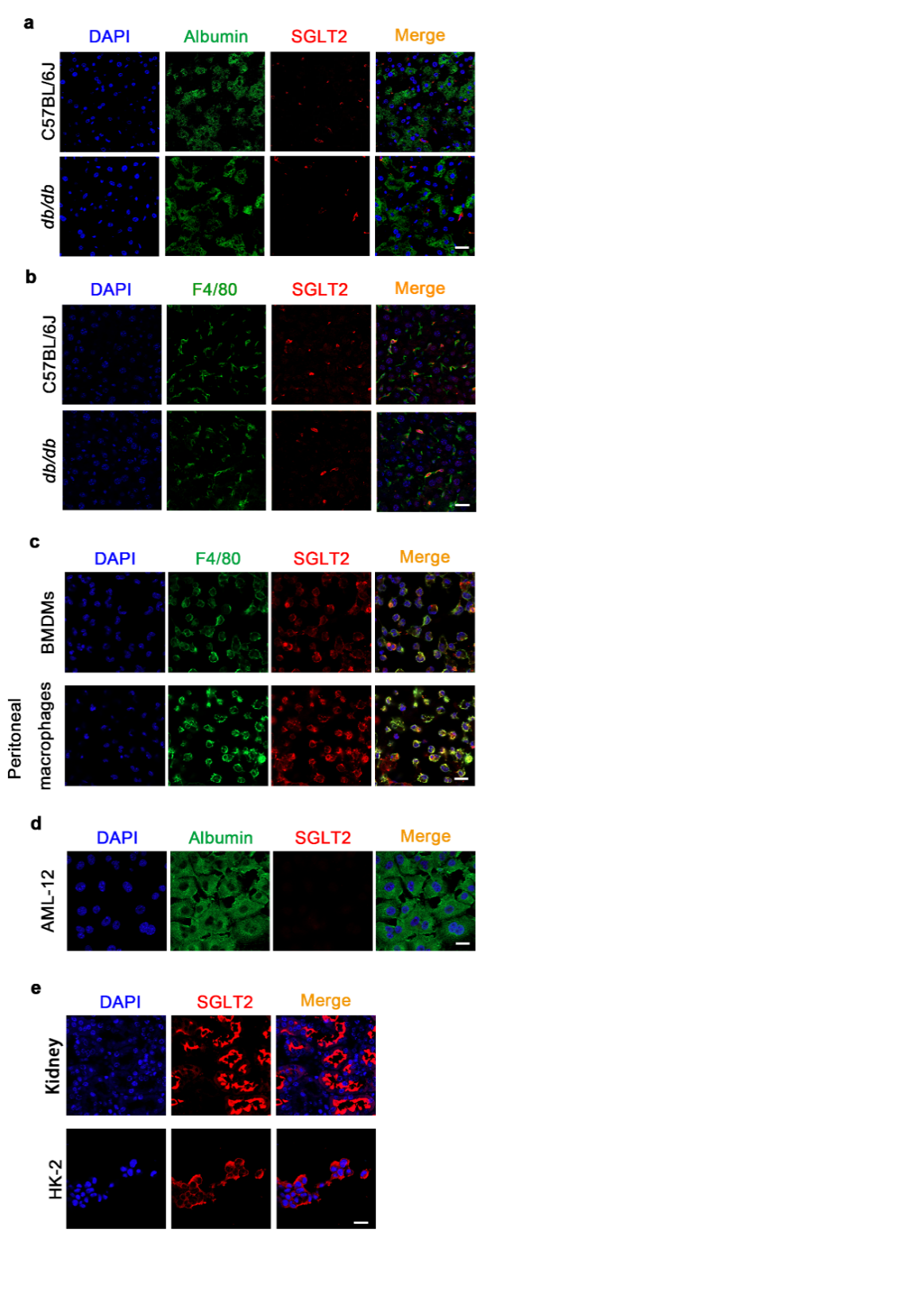
**

**Fig. S4 The expression of SGLT2 protein in macrophages.**

**a** Representative images of immunostaining for sodium-glucose co-transporter 2 (SGLT2) and albumin (hepatocyte marker) in the liver sections of C57BL/6J mice and *db/db* mice. **b** Representative images of immunostaining for SGLT2 and F4/80 (mature macrophage marker) in the liver sections of C57BL/6J mice and *db/db* mice. **c** Representative images of immunostaining for SGLT2 and F4/80 in cell smears of mouse bone marrow-derived macrophages (BMDMs) and peritoneal macrophages. **d** Representative images of immunostaining for SGLT2 and albumin in cell smears of mouse hepatocyte cell line AML-12 cells. **e** Representative images of immunostaining for SGLT2 in the kidney sections of C57BL/6J mice and in cell smears of human kidney proximal tubule epithelial cell line HK-2 cells that both served as positive controls. Scale bar = 20 μm.

**
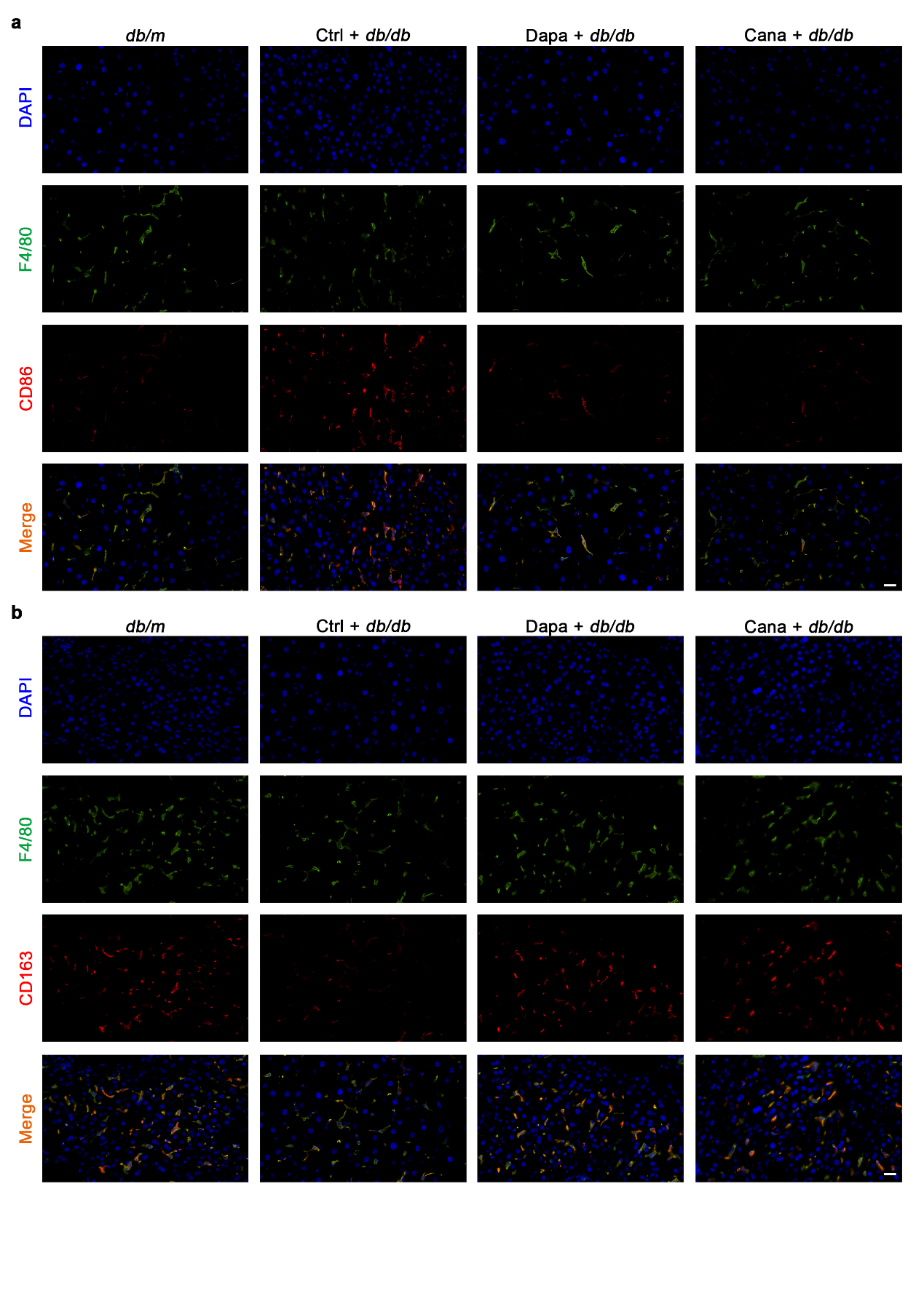
**

**Fig. S5 SGLT2i promotes macrophage polarization from M1 to M2 phenotype in *db/db* mice.**

Eight-week-old male *db/db* mice were treated with dapagliflozin (1 mg·kg^-1^·d^-1^), canagliflozin (10 mg·kg^-1^·d^-1^) or vehicle for 8 weeks. Age-matched male *db/m* mice treated with vehicle were used as normal control. **a** Representative images of macrophages immunostained for F4/80 (mature macrophage marker) and CD86 (M1 marker) in liver sections. **b** Representative images of macrophages immunostained for F4/80 and CD163 (M2 marker) in liver sections. Nuclei were labeled with DAPI (blue). Scale bar = 20 μm. Ctrl, control; Cana, canagliflozin; Dapa, dapagliflozin.

**
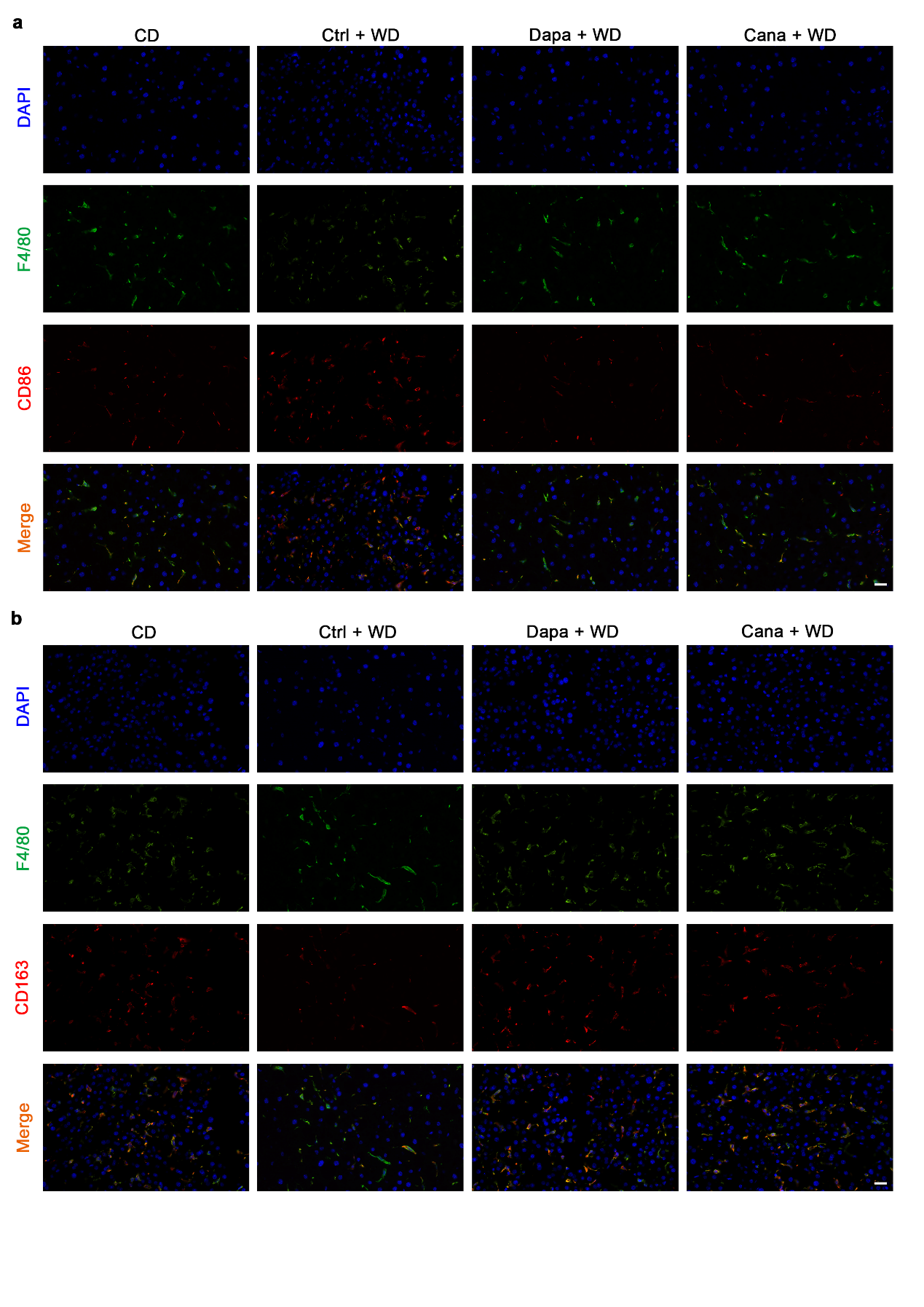
**

**Fig. S6 SGLT2i promotes macrophage polarization from M1 to M2 phenotype in western diet-induced NAFLD mice.**

Six-week-old male C57BL/6J mice were fed on a chow diet (CD) or a western diet (WD) for 20 weeks. Subsequently, the WD-fed mice were treated with dapagliflozin (1 mg·kg^-1^·d^-1^), canagliflozin (10 mg·kg^-1^·d^-1^) or vehicle for 8 weeks. The CD-fed mice treated with vehicle were included as normal control. **a** Representative images of macrophages immunostained for F4/80 (mature macrophage marker) and CD86 (M1 marker) in liver sections. **b** Representative images of macrophages immunostained for F4/80 and CD163 (M2 marker) in liver sections. Nuclei were labeled with DAPI (blue). Scale bar = 20 μm. Ctrl, control; Cana, canagliflozin; Dapa, dapagliflozin.

**
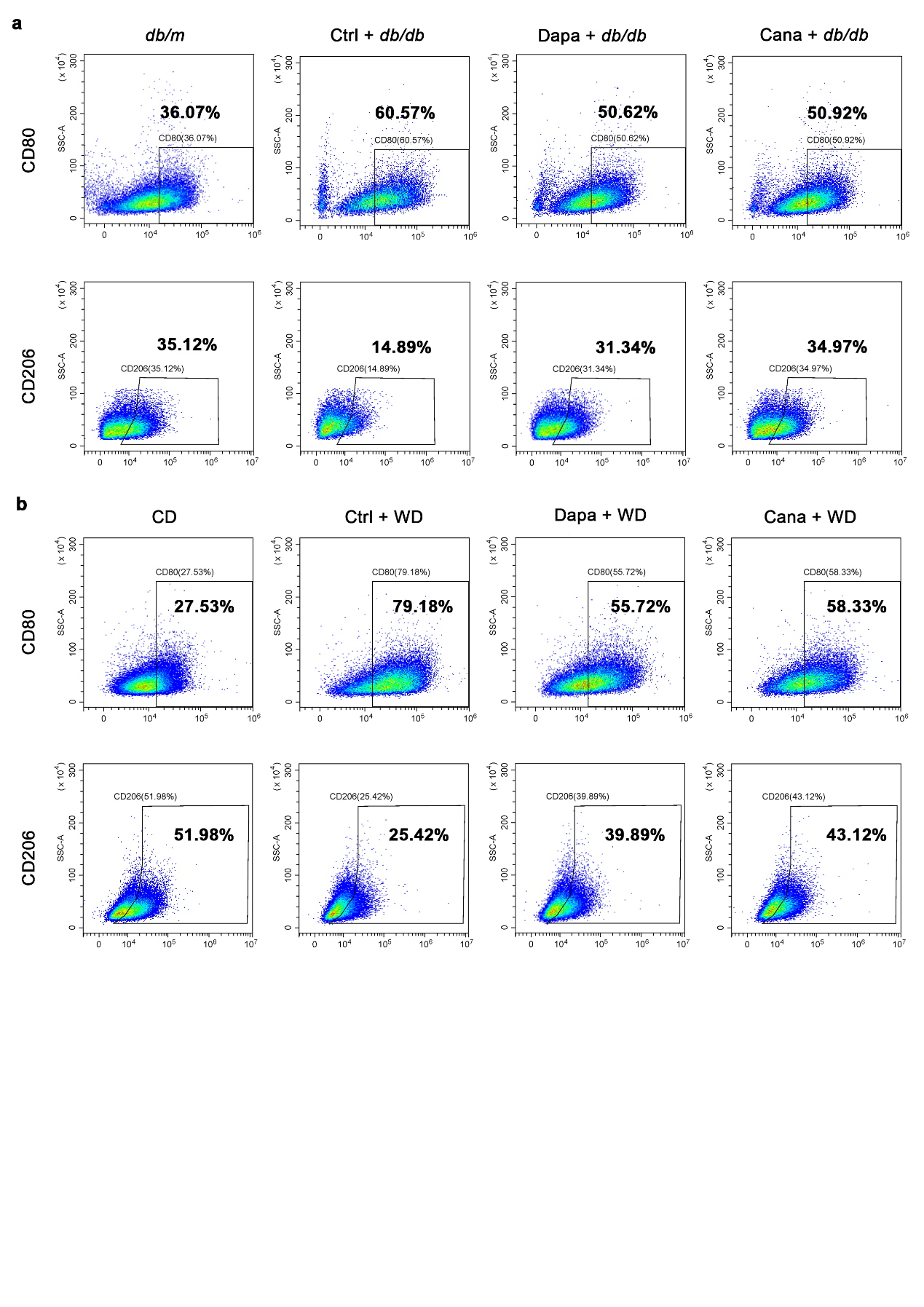
**

**Fig. S7 SGLT2i promotes liver macrophage polarization from M1 to M2 phenotype in two NAFLD models.**

Eight-week-old male *db/db* mice were treated with dapagliflozin (1 mg·kg^-1^·d^-1^), canagliflozin (10 mg·kg^-1^·d^-1^) or vehicle for 8 weeks. Age-matched male *db/m* mice treated with vehicle were used as normal control. Six-week-old male C57BL/6J mice were fed on a chow diet (CD) or a western diet (WD) for 20 weeks. Subsequently, the WD-fed mice were treated with dapagliflozin (1 mg·kg^-1^·d^-1^), canagliflozin (10 mg·kg^-1^·d^-1^) or vehicle for 8 weeks. The CD-fed mice treated with vehicle were included as normal control. Representative images of flow cytometry analysis for CD80 (M1 marker) and CD206 (M2 marker) in mouse liver macrophages isolated from *db/db* mice (**a**) or WD-fed mice (**b**). Ctrl, control; Cana, canagliflozin; Dapa, dapagliflozin.

**
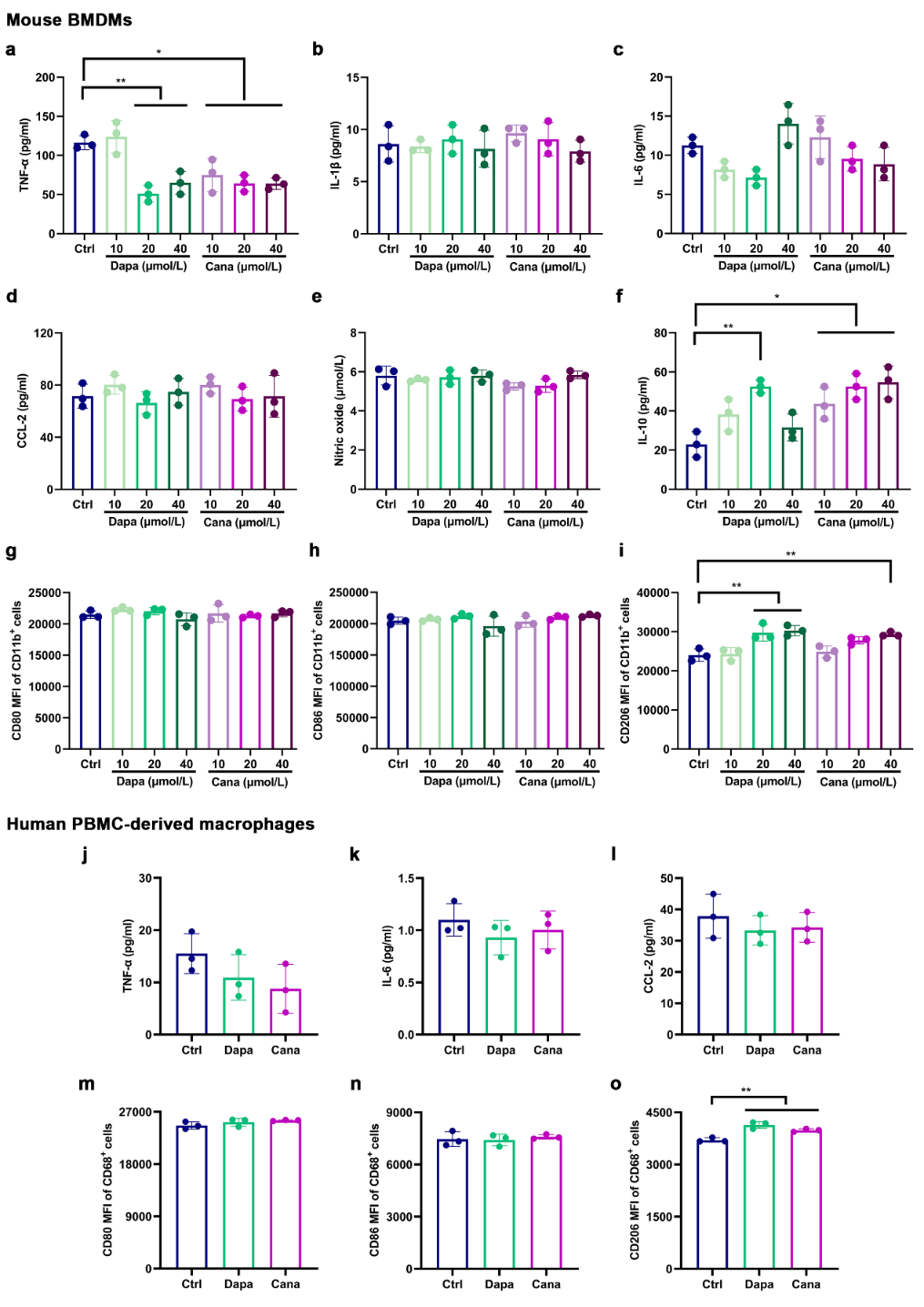
**

**Fig. S8 SGLT2i modulates M1 and M2 macrophage polarization in mouse BMDMs and human PBMC-derived macrophages.**

Mouse bone marrow-derived macrophages (BMDMs) or human peripheral blood mononuclear cell (PBMC)-derived macrophages were cultured for 12 h with different concentrations or 20 µmol/L of dapagliflozin, canagliflozin or vehicle. **a−f** The supernatant levels of pro-inflammatory (**a−e**) and anti-inflammatory (**f**) factors in mouse BMDMs measured by ELISA and chemical assay. **g−i** The mean fluorescence intensity (MFI) of M1 markers CD80 and CD86 (**g**, **h**), and M2 marker CD206 (**i**) in mouse BMDMs detected by flow cytometry. **j−l** The supernatant levels of pro-inflammatory cytokines and chemokines in human PBMC-derived macrophages measured by ELISA. **m−o** The MFI of CD80 and CD86 (**m**, **n**), and CD206 (**o**) in human PBMC-derived macrophages detected by flow cytometry. *n* = 3 per group. Data are expressed as mean ± SD. Statistical analysis was performed by ANOVA followed by the post hoc Tukey-Kramer test. **P*<0.05, ***P*<0.01. Ctrl, control; Cana, canagliflozin; Dapa, dapagliflozin.

**
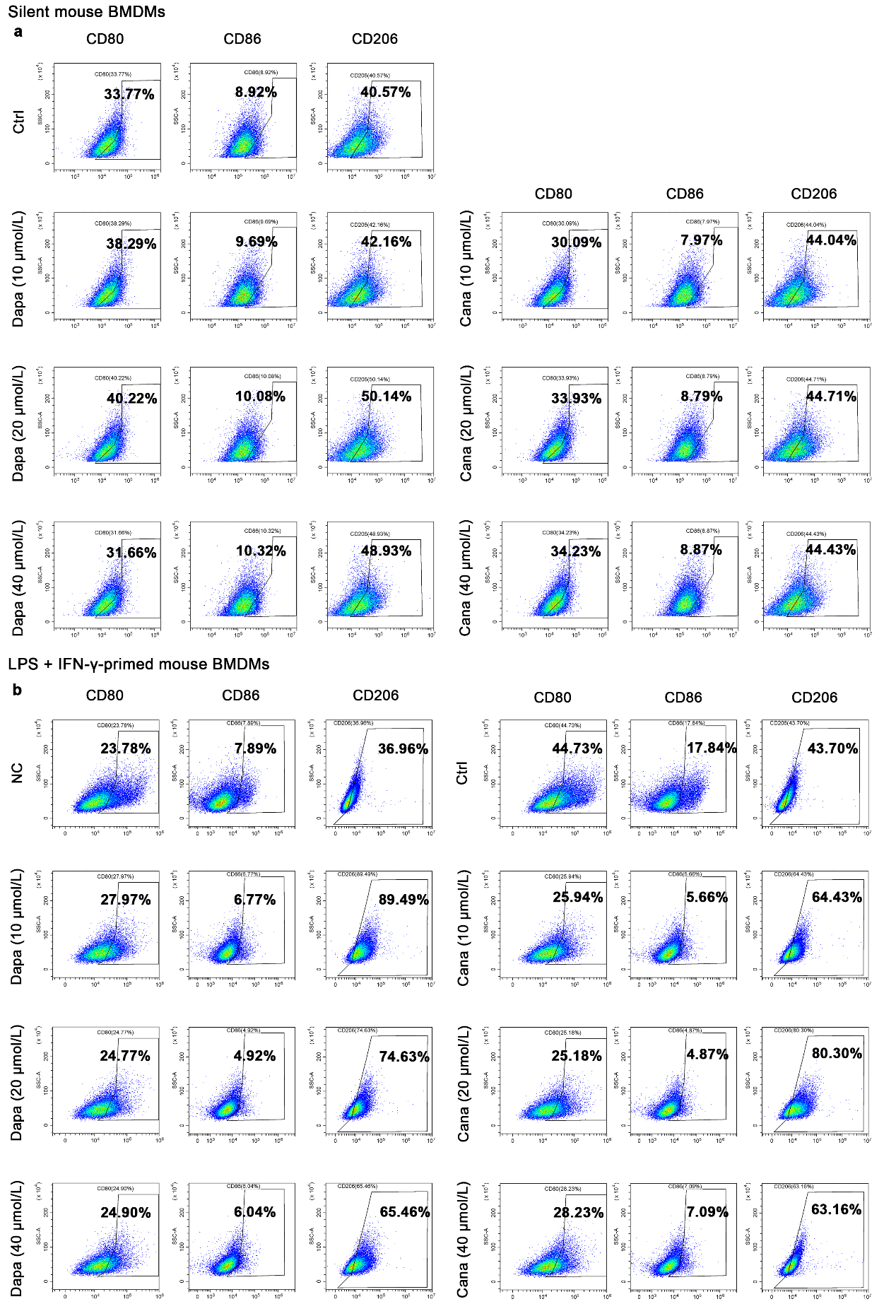
**

**Fig. S9 SGLT2i modulates M1 and M2 macrophage polarization in mouse BMDMs.**

Mouse bone marrow-derived macrophages (BMDMs) were incubated for 12 h with different concentrations of dapagliflozin, canagliflozin or vehicle in the presence or absence of lipopolysaccharide (LPS, 100 ng/mL) + interferon-γ (IFN-γ, 50 ng/mL) that were used for inducing M1 polarization. Representative images of flow cytometry analysis for M1 markers CD80 and CD86, and M2 marker CD206 in silent (**a**) and LPS + IFN-γ-primed (**b**) mouse BMDMs. Ctrl, control; Cana, canagliflozin; Dapa, dapagliflozin; NC, normal control.

**
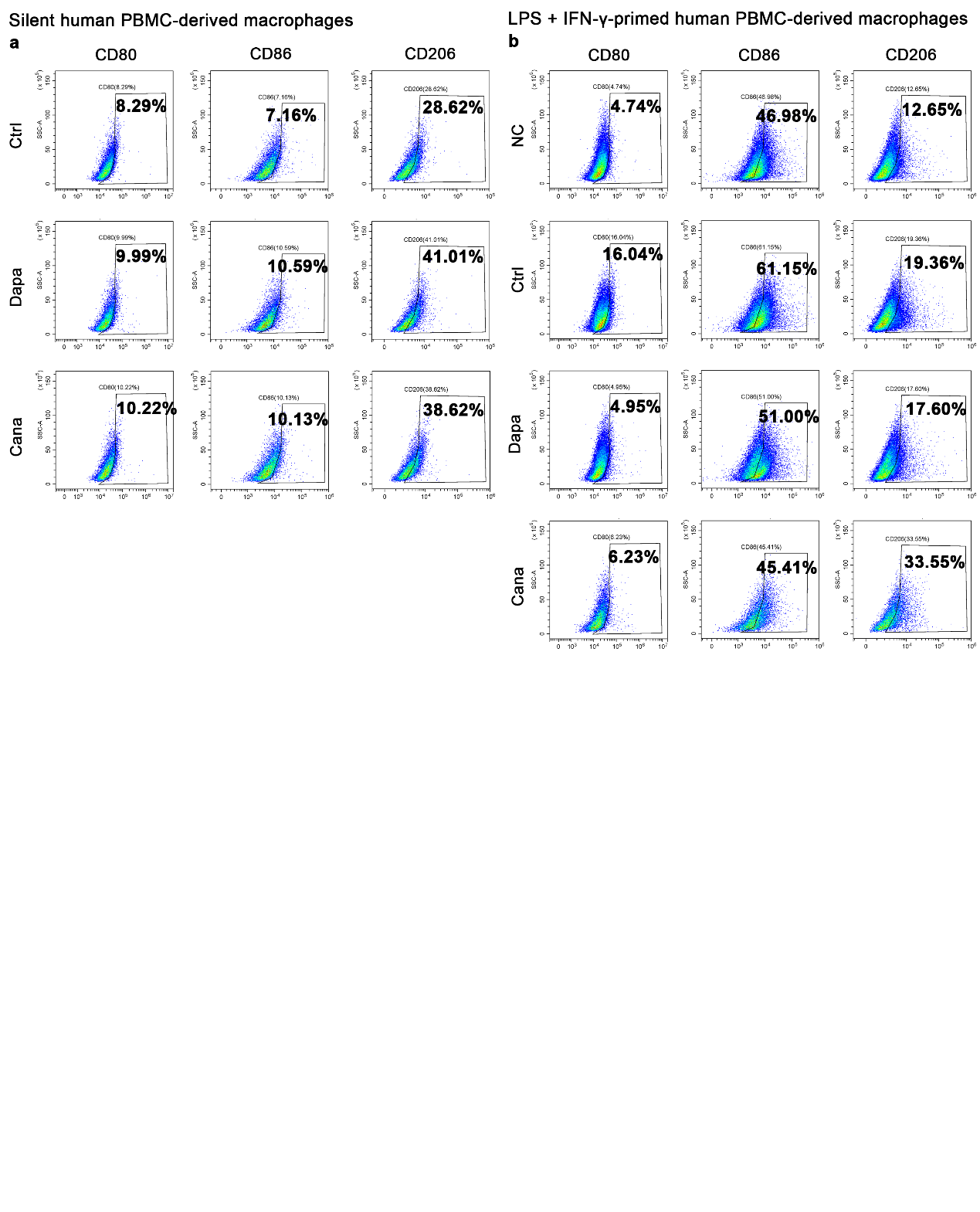
**

**Fig. S10 SGLT2i modulates M1 and M2 macrophage polarization in** **human PBMC-derived macrophages.**

Human peripheral blood mononuclear cell (PBMC)-derived macrophages were incubated for 12 h with 20 µmol/L dapagliflozin, canagliflozin or vehicle in the presence or absence of lipopolysaccharide (LPS, 100 ng/mL) + interferon-γ (IFN-γ, 50 ng/mL) that were used for inducing M1 polarization. Representative images of flow cytometry analysis for M1 markers CD80 and CD86, and M2 marker CD206 in silent (**a**) and LPS + IFN-γ-primed (**b**) human PBMC-derived macrophages. Ctrl, control; Cana, canagliflozin; Dapa, dapagliflozin; NC, normal control.

**
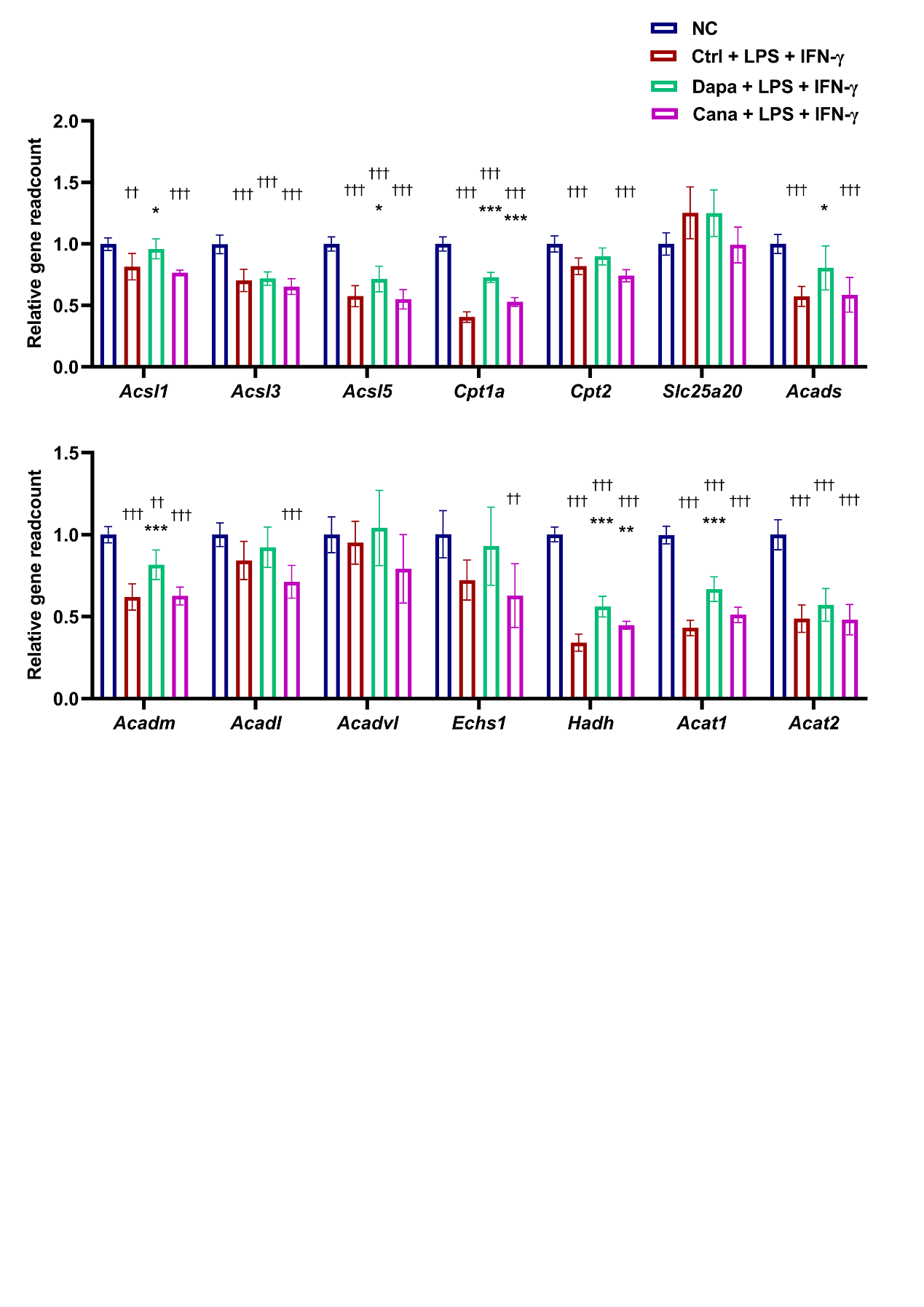
**

**Fig. S11 SGLT2i enhances fatty acid oxidation in M1 macrophages.**

Mouse bone marrow-derived macrophages (BMDMs) were cultured for 12h with 20 µmol/L dapagliflozin, canagliflozin or vehicle in the presence or absence of lipopolysaccharide (LPS, 100 ng/mL) + interferon-γ (IFN-γ, 50 ng/mL) that were used for inducing M1 polarization. Relative readcount of genes related to fatty acid β-oxidation based on RNA-seq data. Data are expressed as mean ± SD. Statistical analysis was performed by ANOVA followed by the post hoc Tukey-Kramer test. **P*<0.05, ***P*<0.01, ****P*<0.001 vs vehicle control-treated group with LPS + IFN-γ; ^††^*P*<0.01, ^†††^*P*<0.001 vs normal control group. Ctrl, control; Cana, canagliflozin; Dapa, dapagliflozin; NC, normal control.

**
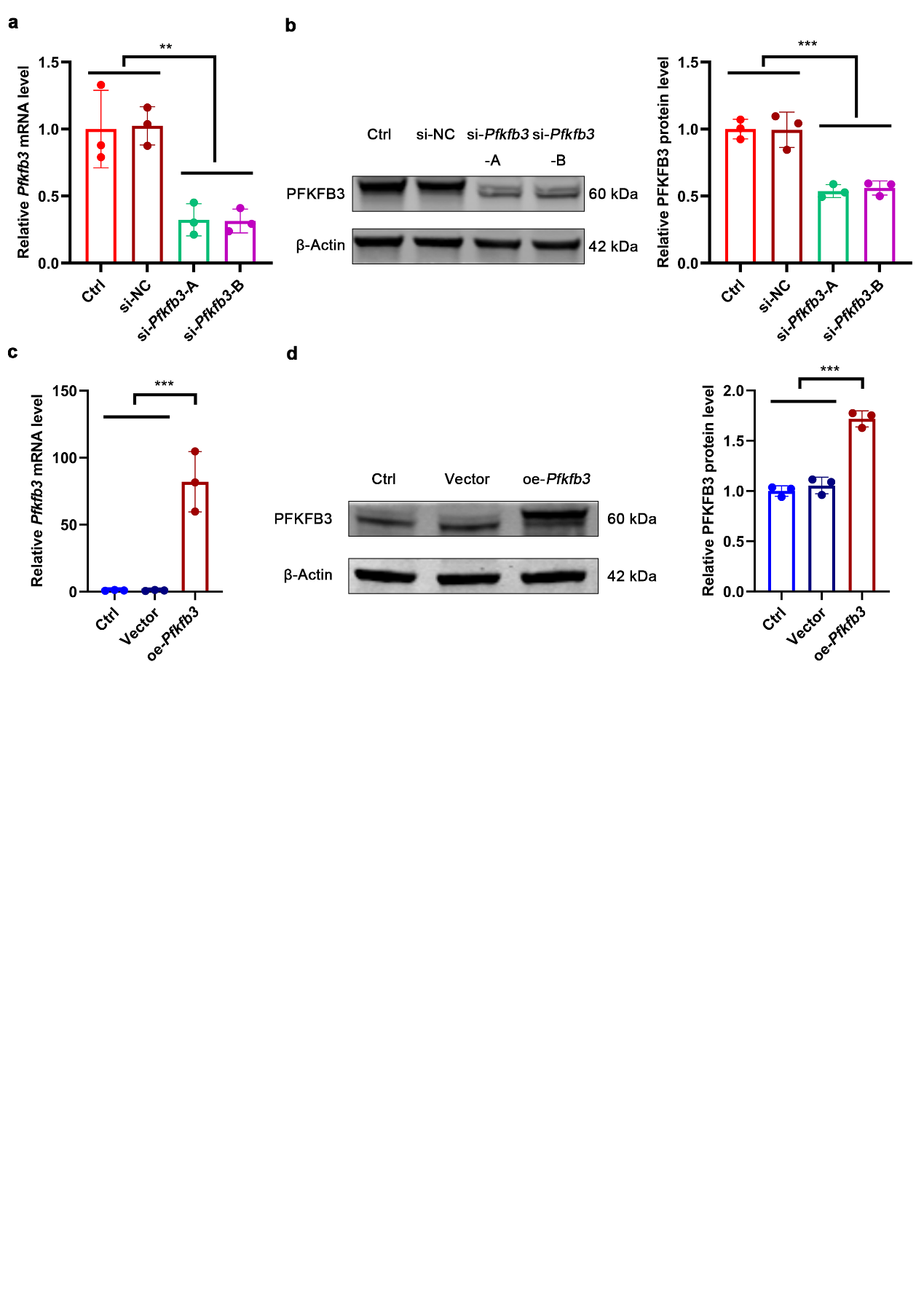
**

**Fig. S12 Knockdown and overexpression of *Pfkfb3* in M1 macrophages.**

**a, b** The mouse macrophage cell line RAW264.7 cells were transfected for 24 h with or without small interfering RNAs (siRNAs), si-NC, si-*Pfkfb3*-A and si-*Pfkfb3*-B, and then incubated for 12 h with LPS that were used for inducing M1 polarization. **c, d** RAW264.7 cells were transfected with or without *Pfkfb3* overexpression plasmid (oe-*Pfkfb3*) and empty vector for 24 h, and then cultured with LPS for 12 h. The PFKFB3 mRNA (**a**, **c**) and protein (**b**, **d**) levels detected by quantitative real-time PCR and Western blot, respectively. *n* = 3 per group. Data are expressed as mean ± SD. Statistical analysis was performed by ANOVA followed by the post hoc Tukey-Kramer test. ***P*<0.01, ****P*<0.001. Ctrl, control; NC, negative control.

**
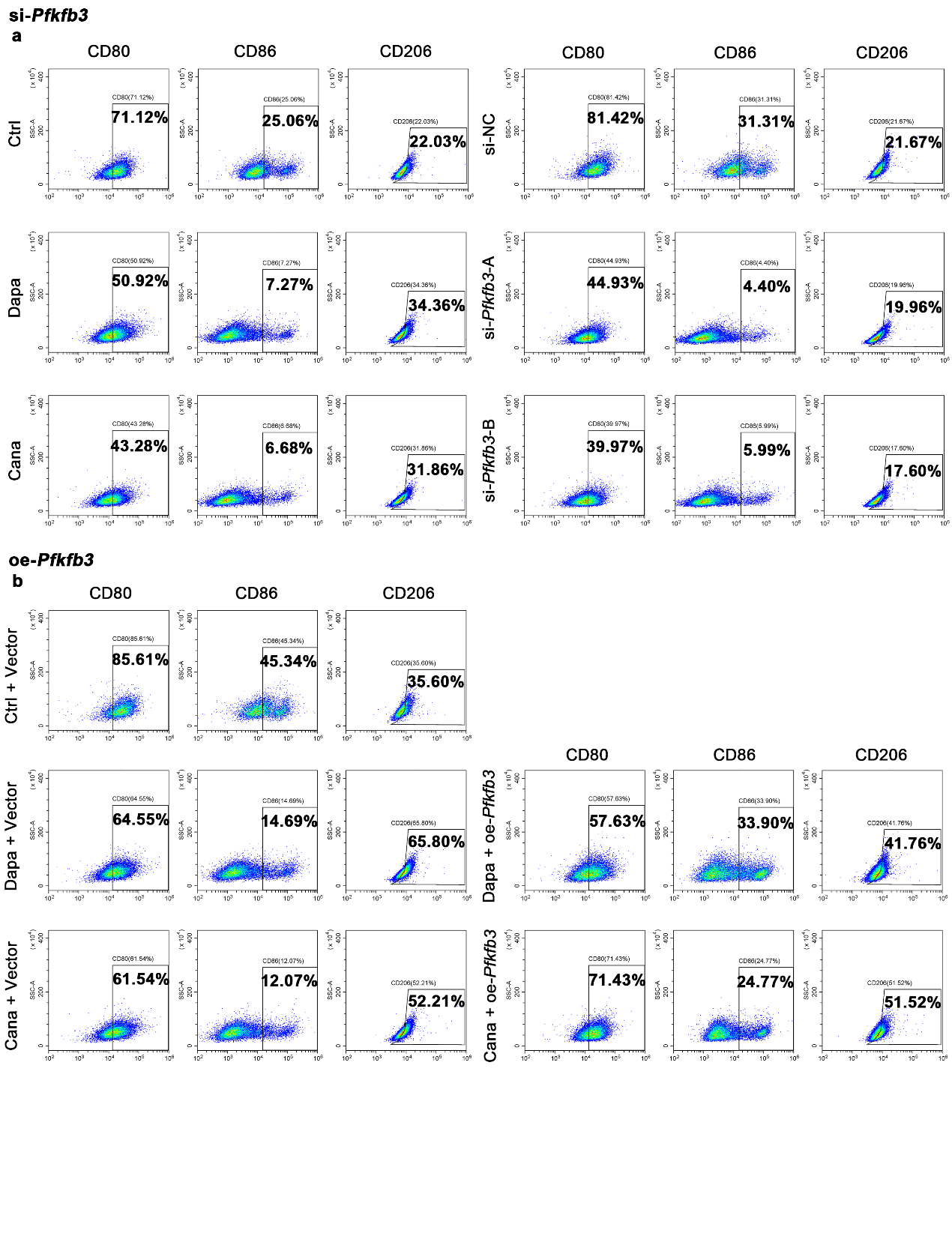
**

**Fig. S13 PFKFB3 is involved in the phenotype shift of macrophages induced by SGLT2i.**

**a** The mouse macrophage cell line RAW264.7 cells were cultured for 12 h with 20 µmol/L dapagliflozin, canagliflozin or vehicle in the presence of lipopolysaccharide (LPS, 100 ng/mL) that were used for inducing M1 polarization. The cells were transfected for 24 h with small interfering RNAs (siRNAs), si-NC, si-*Pfkfb3*-A and si-*Pfkfb3*-B, and then incubated with LPS for 12 h. **b** RAW264.7 cells were transfected with *Pfkfb3* overexpression plasmid (oe-*Pfkfb3*) or empty vector for 24 h, and then cultured with LPS and 20 µmol/L dapagliflozin, canagliflozin or vehicle for 12 h. Representative images of flow cytometry analysis for M1 markers CD80 and CD86, and M2 marker CD206. *n* = 3 per group. Ctrl, control; Cana, canagliflozin; Dapa, dapagliflozin; NC, negative control.

**
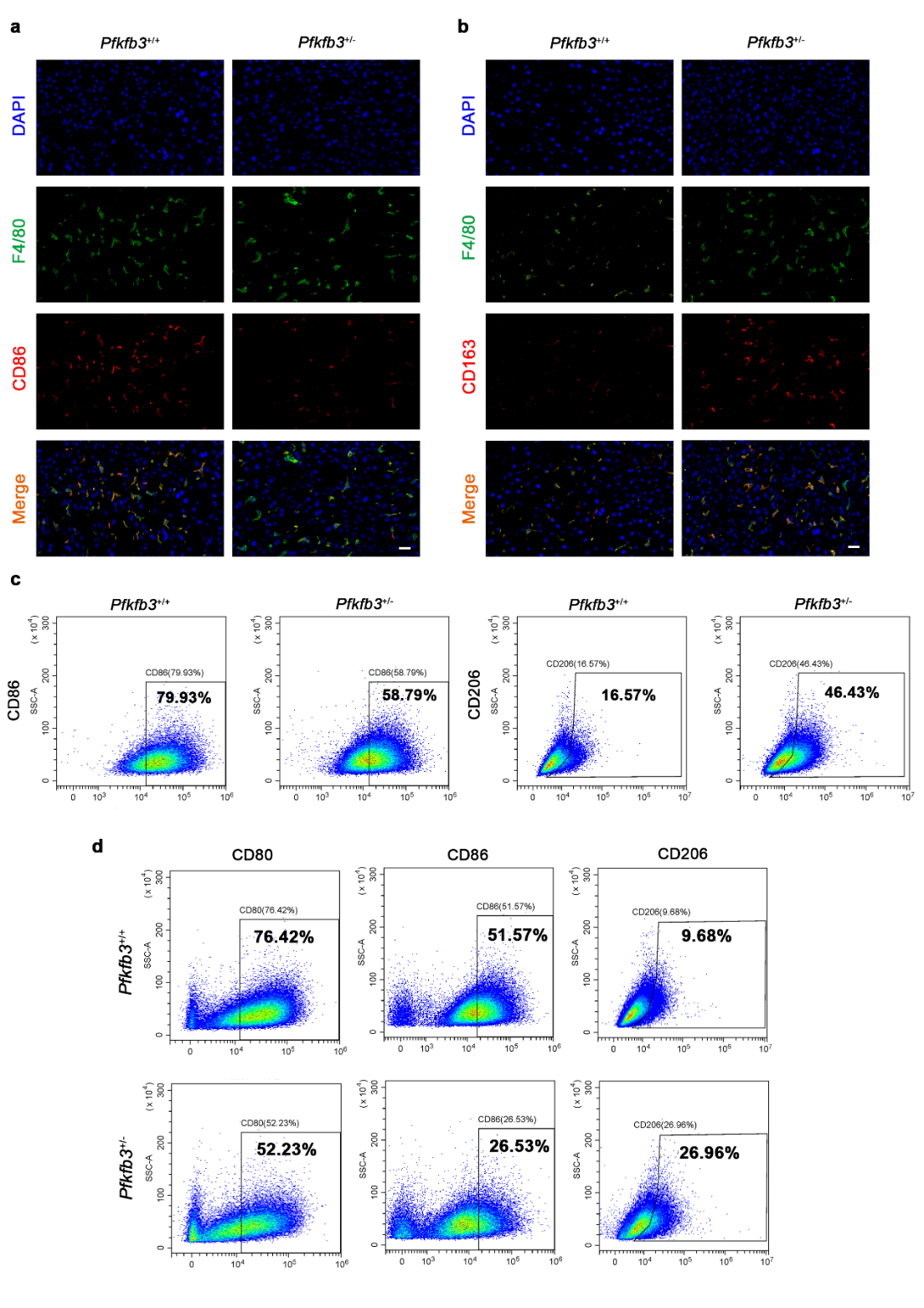
**

**Fig. S14 *Pfkfb3* knockout promotes macrophage polarization from M1 to M2 phenotype.**

Six-week-old male global heterozygous *Pfkfb3* knockout (*Pfkfb3^+/−^*) mice and littermate *Pfkfb3^+/+^* mice were fed on a western diet (WD) for 12 weeks. **a, b** Representative images of macrophages immunostained for F4/80 (mature macrophage marker) and CD86 (M1 marker) (**a**) or CD163 (M2 marker) (**b**) in liver sections. Nuclei were labeled with DAPI (blue). Scale bar = 20 μm. **c** Representative images of flow cytometry analysis for CD86 and CD206 (another M2 marker) in isolated mouse liver macrophages. *n* = 4 per group. BMDMs were isolated from six-week-old male *Pfkfb3^+/−^* and *Pfkfb3^+/+^* mice, and were incubated for 12 h with lipopolysaccharide (LPS, 100 ng/mL) + interferon-γ (IFN-γ, 50 ng/mL) that were used for inducing M1 polarization. **d** Representative images of flow cytometry analysis for CD80 (another M1 marker), CD86 and CD206. *n* = 4 per group.

**
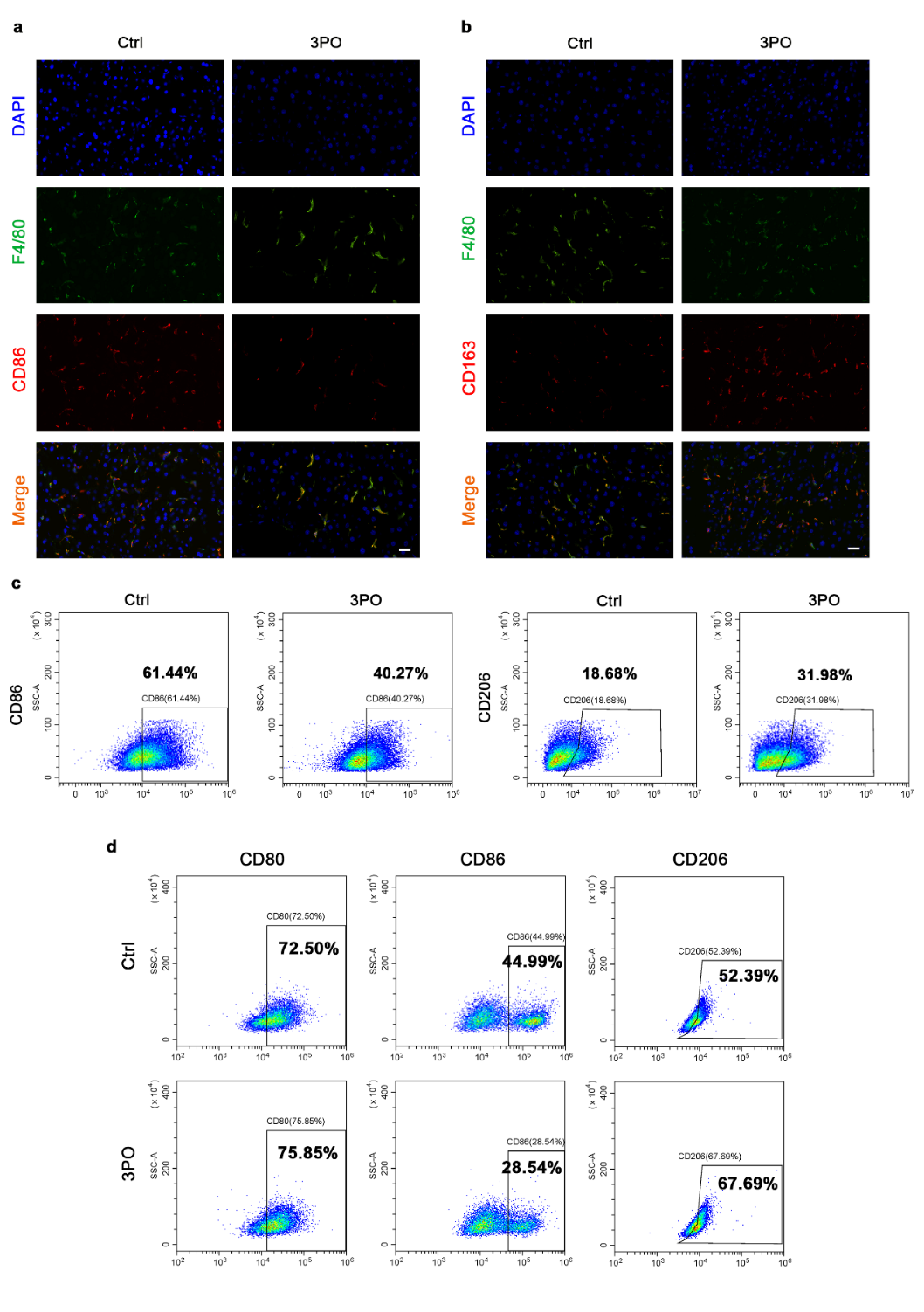
**

**Fig. S15 Specific inhibition of PFKFB3 promotes macrophage polarization from M1 to M2 phenotype.**

Six-week-old male C57BL/6J mice were fed on a western diet (WD) for 8 weeks, and were treated for 4 weeks with the PFKFB3-specific inhibitor 3-(3-pyridinyl)-1-(4-pyridinyl)-2-propen-1-one (3PO; 50 mg/kg, 4 times every week) or vehicle. **a, b** Representative images of macrophages immunostained for F4/80 (mature macrophage marker) and CD86 (M1 marker) (**a**) or CD163 (M2 marker) (**b**) in liver sections. Nuclei were labeled with DAPI (blue). Scale bar = 20 μm. **c** Representative images of flow cytometry analysis for CD86 and CD206 (another M2 marker) in isolated mouse liver macrophages. *n* = 4 per group. BMDMs isolated from C57BL/6J mice were incubated for 12 h with 10 μmol/L 3PO or vehicle in the presence of lipopolysaccharide (LPS, 100 ng/mL) + interferon-γ (IFN-γ, 50 ng/mL) that were used for inducing M1 polarization. **d** Representative images of flow cytometry analysis for CD80 (another M1 marker), CD86 and CD206. *n* = 3 per group. Ctrl, control.

SUPPLEMENTARY TABLES

**Table S1. The kits used in this study**

| **Reagent** | **Source** | **Application** | **Identifier** |
| --- | --- | --- | --- |
| TG detection kit | Applygen | Biochemical assay | Cat#: E1025 |
| TC detection kit | Applygen | Biochemical assay | Cat#: E1026 |
| FC detection kit | Applygen | Biochemical assay | Cat#: E1027 |
| Nitric oxide detection kit | Beyotime | Chemical assay | Cat#: S0021S |
| Mouse insulin ELISA Kit | Alpco | ELISA | Cat#: 80-INSMSU-E01  RRID: AB_2792981 |
| Mouse TNF-α ELISA Kit | Invitrogen | ELISA | Cat#: 88-7324  RRID: AB_2575076 |
| Mouse IL-1β ELISA kit | Invitrogen | ELISA | Cat#: 88-7013  RRID: AB_2574943 |
| Mouse IL-6 ELISA kit | Invitrogen | ELISA | Cat#: 88-7064  RRID: AB_2574986 |
| Mouse IL-10 ELISA kit | Invitrogen | ELISA | Cat#: 88-7105  RRID: AB_2575000 |
| Mouse CCL-2 ELISA kit | Invitrogen | ELISA | Cat#: 88-7391  RRID: AB_2575113 |
| Human/mouse TGF-β ELISA kit | Invitrogen | ELISA | Cat#: 88-8350  RRID: AB_2491100 |
| Human TNF-α ELISA kit | Invitrogen | ELISA | Cat#: 88-7346  RRID: AB_2575097 |
| Human IL-6 ELISA kit | Invitrogen | ELISA | Cat#: 88-7066  RRID: AB_2574994 |
| Human CCL-2 ELISA kit | Invitrogen | ELISA | Cat#: 88-7399  RRID: AB_2575114 |
| H&E staining kit | Solarbio | Histopathological assay | Cat#: G1120 |
| Masson staining kit | Solarbio | Histopathological assay | Cat#: G1340 |
| Sirius red staining kit | Solarbio | Histopathological assay | Cat#: G1472 |
| Oil red O staining kit | Solarbio | Histopathological assay | Cat#: G1260 |
| Seahorse XF glycolytic rate assay kit | Agilent Technologies | Seahorse analysis | Cat#: 103344-100 |
| Seahorse XF cell Mito stress test kit | Agilent Technologies | Seahorse analysis | Cat#: 103015-100 |
| MojoSort™ human CD14 selection kit | Biolegend | Magnetic bead sorting | Cat#: 480025 |

TG, triglyceride; TC, total cholesterol; FC, free cholesterol.

**Table S2. The antibodies used in this study**

| **Reagent** | **Source** | **Application** | **Identifier** |
| --- | --- | --- | --- |
| Anti-F4/80 antibody | Abcam | IF (1:100) | Cat#: ab6640  RRID: AB_1140040 |
| Anti-F4/80 antibody | Bio-rad | IF (1:100) | Cat#: MCA497G  RRID: AB_872005 |
| SGLT2 antibody (D-6) | Santa cruz Biotechnology | IF (1:100) | Cat#: sc-393350  RRID: AB_2814658 |
| Anti-SGLT2 antibody | Abcam | IF (1:100) | Cat#: ab306558 |
| Anti-albumin antibody | Abcam | IF (1:100) | Cat#: ab207327  RRID: AB_2755031 |
| Anti-rat IgG (H + L), (Alexa Fluor® 488 Conjugate) | Cell Signaling Technology | IF (1:400) | Cat#: 4416  RRID:AB_10693769 |
| Anti-mouse IgG (H + L), F(ab')2 Fragment (Alexa Fluor® 594 Conjugate) | Cell Signaling Technology | IF (1:400) | Cat#: 8890  RRID: AB_2714182 |
| Anti-rabbit IgG (H + L), F(ab')2 Fragment (Alexa Fluor® 594 Conjugate) | Cell Signaling Technology | IF (1:400) | Cat#: 8889  RRID: AB_2716249 |
| Anti-CD163 antibody [EPR19518] | Abcam | IF (1:100) | Cat#: ab182422  RRID: AB_2753196 |
| Anti-CD86 antibody [EPR21962] | Abcam | IF (1:100) | Cat#: ab239075  RRID: AB_2927417 |
| FITC anti-mouse F4/80 antibody | Biolegend | FC (1:100) | Cat#:123108  RRID: AB_893502 |
| PerCP/Cyanine5.5 anti-mouse/human CD11b antibody | Biolegend | FC (1:100) | Cat#: 101228  RRID: AB_893232 |
| APC anti-mouse CD80 antibody | Biolegend | FC (1:100) | Cat#: 104714  RRID: AB_313135 |
| PE/Cyanine7 anti-mouse CD86 antibody | Biolegend | FC (1:100) | Cat#: 105014  RRID: AB_439783 |
| Brilliant Violet 421™ anti-mouse CD206 (MMR) antibody | Biolegend | FC (1:50) | Cat#: 141717  RRID: AB_2562232 |
| PE/Cyanine7 anti-human CD68 antibody | Biolegend | FC (1:50) | Cat#: 333816  RRID: AB_2562936 |
| FITC anti-human CD80 antibody | Biolegend | FC (1:100) | Cat#: 305206  RRID: AB_314502 |
| APC anti-human CD86 antibody | Biolegend | FC (1:100) | Cat#: 305412  RRID: AB_493231 |
| PE anti-human CD206 (MMR) antibody | Biolegend | FC (1:100) | Cat#: 321106  RRID: AB_571910 |
| Anti-SREBP1 antibody | Abcam | WB (1:1000) | Cat#: ab28481  RRID: AB_778069 |
| Anti-fatty acid synthase antibody | Abcam | WB (1:1000) | Cat#: ab22759  RRID: AB_732316 |
| Anti-acetyl coenzyme A carboxylase antibody | Abcam | WB (1:1000) | Cat#: ab45174  RRID: AB_867475 |
| Anti-PFKFB3 antibody [EPR12594] | Abcam | WB (1:1000) | Cat#: ab181861  RRID: AB_3095816 |
| Anti-β-Actin (BA3R) mouse mAb | Applygen | WB (1:5000) | Cat#: C1313  RRID: AB_3076668 |
| IRDye 800CW goat (polyclonal) anti-rabbit IgG (H+L) antibody | LI-COR Biosciences | WB (1:10,000) | Cat#: 925-32211  RRID: AB_2651127 |
| IRDye 800CW goat (polyclonal) anti-mouse IgG (H+L) antibody | LI-COR Biosciences | WB (1:10,000) | Cat#: 925-32210  RRID: AB_2687825 |

IF, immunofluorescence; FC, flow cytometry; WB, Western blot.

**Table S3. Multiple reaction monitoring parameters for compounds**

| **Compound** | **Parent (m/z)** | **Daughter (m/z)** | **Cone (V)** | **Collision energy (v)** | **Mode** |
| --- | --- | --- | --- | --- | --- |
| Phosphoenolpyruvate | 169.07 | 151.06 | 30 | 8 | ESI+ |
| Glyceraldehyde-3-P | 171.00 | 98.94 | 30 | 8 | ESI+ |
| 3-Phosphoglyceric acid | 187.06 | 99.07 | 30 | 9 | ESI+ |
| Glucose-6-P/Fructose-6-P | 261.17 | 99.13 | 40 | 10 | ESI+ |
| Fructose-1,6-diphosphate | 341.10 | 127.00 | 10 | 20 | ESI+ |
| Acetyl coenzyme A | 810.31 | 303.20 | 16 | 34 | ESI+ |
| Pyruvic acid | 86.97 | 42.97 | 40 | 10 | ESI− |
| Lactic acid | 88.86 | 42.81 | 10 | 7 | ESI− |
| Fumaric acid | 114.97 | 71.02 | 28 | 10 | ESI− |
| Succinic acid | 117.01 | 73.06 | 16 | 10 | ESI− |
| Oxaloacetic acid | 131.02 | 87.08 | 28 | 10 | ESI− |
| Malic acid | 133.02 | 115.06 | 10 | 10 | ESI− |
| α-Ketoglutarate | 145.04 | 101.08 | 16 | 10 | ESI− |
| Aconitic acid | 174.84 | 86.87 | 34 | 15 | ESI− |
| Citric acid | 191.09 | 111.07 | 4 | 10 | ESI− |

ESI, electrospray ionization

**Table S4. siRNA sequences against mouse *Pfkfb3* gene used in this study**

| **Dulex name** | **Sense (5'−3')** | **Antisense (5'−3')** |
| --- | --- | --- |
| si-NC | UUCUCCGAACGUGUCACGUTT | ACGUGACACGUUCGGAGAATT |
| si-*Pfkfb3*-A | GCGAGAAUGAGUACAACUUTT | AAGUUGUACUCAUUCUCGCTT |
| si-*Pfkfb3*-B | GGAUAGGUGUUCCAACGAATT | UUCGUUGGAACACCUAUCCTT |

NC, negative control.

**Table S5. The primers for quantitative real-time PCR used in this study**

| **Gene ID** | **Gene symbol** | **Gene name** | **Primer sequence 5’−3’** | **Tm (˚C)** | **Product length (bp)** |
| --- | --- | --- | --- | --- | --- |
| 14080 | *Fabp1* | fatty acid binding protein 1, liver | F: TGGTCCGCAATGAGTTCACCCT  R: CCAGCTTGACGACTGCCTTGACTT | 64 | 84 |
| 12491 | *Cd36* | CD36 molecule | F: GACTGGGACCATTGGTGATGA  R: AAGGCCATCTCTACCATGCC | 60 | 90 |
| 20787 | *Srebf1* | sterol regulatory element binding transcription factor 1 | F: CACTTCTGGAGACATCGCAAAC  R: ATGGTAGACAACAGCCGCATC | 61 | 282 |
| 14104 | *Fasn* | fatty acid synthase | F: CTGCGGAAACTTCAGGAAATG  R: GGTTCGGAATGCTATCCAGG | 58 | 301 |
| 107476 | *Acaca* | acetyl-coenzyme A carboxylase alpha | F: GGCCAGTGCTATGCTGAGAT  R: AGGGTCAAGTGCTGCTCCA | 61 | 108 |
| 19013 | *Ppara* | peroxisome proliferator activated receptor alpha | F: TATTCGGCTGAAGCTGGTGTAC  R: CTGGCATTTGTTCCGGTTCT | 60 | 76 |
| 19016 | *Pparg* | peroxisome proliferator activated receptor gamma | F: ATTCTGGCCCACCAACTTCGG  R: TGGAAGCCTGATGCTTTATCCCCA | 64 | 339 |
| 12894 | *Cpt1a* | carnitine palmitoyltransferase 1a, liver | F: AGGACCCTGAGGCATCTATT  R: ATGACCTCCTGGCATTCTCC | 59 | 301 |
| 20249 | *Scd1* | stearoyl-coenzyme A desaturase 1 | F: TTCTTGCGATACACTCTGGTGC  R: CGGGATTGAATGTTCTTGTCGT | 61 | 98 |
| 12842 | *Col1a1* | collagen, type I, alpha 1 | F: GCTCCTCTTAGGGGCCACT  R: CCACGTCTCACCATTGGGG | 61 | 103 |
| 11475 | *Acta2* | actin alpha 2, smooth muscle, aorta | F: GTCCCAGACATCAGGGAGTAA  R: TCGGATACTTCAGCGTCAGGA | 61 | 102 |
| 18787 | *Serpine1* | serine (or cysteine) peptidase inhibitor, clade E, member 1 | F: TTCAGCCCTTGCTTGCCTC  R: ACACTTTTACTCCGAAGTCGGT | 61 | 116 |
| 14219 | *Ccn2* | cellular communication network factor 2 | F: GGGCCTCTTCTGCGATTTC  R: ATCCAGGCAAGTGCATTGGTA | 60 | 151 |
| 170768 | *Pfkfb3* | 6-phosphofructo-2-kinase/fructose-2,6-biphosphatase 3 | F: GCCATACCTGAAATGTCCGC  R: GCTCTTCATGTTCTCTGACCTC | 59 | 133 |
| 11461 | *Actb* | actin, beta | F: TGTACCCAGGCATTGCTGAC  R: CTGCTGGAAGGTGGACAGTG | 61 | 149 |
